# Optimization of linear attenuation coefficients and characterization of mechanical and thermal properties in silica ash-reinforced PDMS composites

**DOI:** 10.1038/s41598-026-58992-z

**Published:** 2026-06-29

**Authors:** Maged Mostafa, S. S. Ibrahim, Sherif A. Khairy, Ahmed M. El-khatib

**Affiliations:** 1 Physics Department, Faculty of Science, Cairo University, Giza, Egypt; 2 Physics Department, Faculty of Science, Alexandria University, Alexandria, 21511 Egypt

**Keywords:** Gamma-ray attenuation, Silicone rubber composites, Silica ash, Linear attenuation coefficient, NIST XCOM, Half-value layer, Mechanical reinforcement, Thermal stability, Eco-friendly shielding materials, Engineering, Materials science, Nanoscience and technology

## Abstract

This investigation engineers multifunctional polydimethylsiloxane (PDMS) composites incorporating 0–50 wt% silica ash a valorized industrial byproduct as lightweight, sustainable gamma-ray shielding elastomers. Narrow-beam attenuation experiments spanning diagnostic-to-isotope energies (59.5-1332.5 keV) quantify a monotonic enhancement in linear attenuation coefficients (µ), escalating from 0.3011 cm⁻¹ (pure PDMS) to 0.3651 cm⁻¹ (50 wt% ash) at 59.5 keV, with concomitant reductions in half-value layer (HVL) from 2.30 cm to 1.90 cm. This stems from a paradigm shift in photon interaction physics: photoelectric dominance (< 100 keV) amplifies via elevated effective atomic number (Z_eff_), transitioning to density-mediated Compton scattering (≥ 661.66 keV), validated by < 3.76% deviation from NIST XCOM photon cross-sections. Lead-equivalence analysis reveals the 50 wt% composite demands merely 10–14× lead thickness in the Compton regime, underscoring superior mass efficiency. Hierarchical microstructural analysis (SEM, EDX, XRF) elucidate homogeneous nanofiller dispersion and robust siloxane-filler interfacial coupling, driving progressive stiffening (Young’s modulus: 0.026 to 0.157 MPa at 40 wt%). Optimal mechano-elastic performance manifests at 15 wt% (tensile strength: 0.357 MPa; toughness: 0.591 MJ·m⁻³), beyond which agglomeration induces embrittlement. Thermogravimetric profiles reveal an initial stabilization peak at 10 wt% ash, followed by catalytic depolymerization at higher loadings, rationalized by Lewis acid-base interactions accelerating Si-O bond scission despite augmented char residue. These composites (optimized at 10–20 wt%) exhibit enhanced radiation attenuation together with improved mechanical resilience and elastomeric flexibility, demonstrating their potential for flexible radiation shielding applications.

**Supplementary Information:**

The online version contains supplementary material available at 10.1038/s41598-026-58992-z.

## Introduction

The widespread use of ionizing radiation in medical diagnostics, radiotherapy, nuclear energy, industrial inspection, and space technologies has increased the demand for advanced gamma-ray shielding materials that combine high attenuation efficiency with mechanical flexibility and environmental sustainability. Conventional shielding materials such as lead and other heavy metals exhibit excellent attenuation performance; however, their high density, toxicity, rigidity, and poor processability limit their application in lightweight and flexible systems^1–4^. These limitations have stimulated growing interest in polymer-based radiation shielding composites as safer and more versatile alternatives^5–7^.

Polymeric matrices offer intrinsic advantages including low density, corrosion resistance, ease of fabrication, and mechanical flexibility; however, their low effective atomic number ( Z_eff_) and density result in inherently poor gamma-ray attenuation when used alone^8,9^. To address this limitation, polymer composites incorporating inorganic fillers have been extensively investigated, where shielding performance is strongly influenced by filler chemistry, loading fraction, particle size, and dispersion within the polymer matrix^10–13^. In particular, the incorporation of high-Z fillers such as PbO, Bi₂O₃, WO₃, and tungsten-based compounds has been shown to markedly enhance gamma-ray attenuation, especially at low photon energies dominated by photoelectric absorption processes^14–17^. Nevertheless, the toxicity, high cost, and environmental concerns associated with these fillers motivate the search for sustainable and eco-friendly alternatives^18–21^.

Furthermore, the incorporation of inorganic fillers like zinc oxide into the polydimethylsiloxane (PDMS) matrix has been shown to effectively enhance mass attenuation coefficients while significantly reducing the half-value layer (HVL) and mean free path (MFP) for gamma-ray shielding applications^22^.

Among polymer matrices, silicone rubber emerged as a promising candidate for radiation shielding applications due to its outstanding thermal stability, chemical resistance, flexibility over wide temperature range, and superior aging resistance compared to conventional elastomers^23–26^. Silicone rubber composites reinforced with heavy metal oxide have demonstrated significant improvements in linear and mass attenuation coefficients, half-value layer (HVL), and mean free path (MFP) across a broad gamma-ray energy spectrum^27–30^. However, most reported systems continue to rely on dense and hazard fillers, limiting their scalability and long-term sustainability.

Gamma-ray attenuation in composite materials is governed by energy-dependent interaction mechanisms. At low photon energies (E < 100 keV), photoelectric absorption predominates and shows a strong dependence on the effective atomic number (Z⁴-Z⁵), making filler composition a critical factor^8,10–12^. In the intermediate energy range (≈ 100 keV- 1 MeV), Compton scattering becomes the prevailing interaction mechanism, with attenuation governed primarily by electron density rather than atomic number^7,11,31^. At higher energies, pair production may contribute marginally, although its influence remains limited within the energy range of commonly used laboratory gamma sources^10^. Accordingly, tailoring filler composition and concentration provides a viable pathway to engineer polymer composites with optimized, energy-dependent shielding performance^15,19^.

In parallel with radiation shielding efficiency, mechanical integrity is a key requirement for polymer-based shielding materials, particularly for flexible and wearable applications. Silicone rubber inherently exhibits low Young’s modulus and tensile strength, which can limit its structural performance^32^. Numerous studies have shown that silica-based fillers significantly enhance the mechanical properties of silicone rubber through improved filler-matrix interactions, restricted polymer chain mobility, and the formation of mechanically stiffness interphase regions^33–36^. Both micro- and nano-silica fillers increase Young’s modulus, tensile strength, and hardness, although excessive filler loading can induce agglomeration and reduce elongation at break^37–39^.Waste-derived silica sources, such as fly ash and rice husk ash, offer reinforcement efficiencies comparable to commercial silica while providing significant environmental and economic advantages. Silica ash, a by-product rich in SiO₂ with minor oxide constituents, has been widely utilized as a reinforcing filler in rubber systems^40,41^.

However, its potential as a functional gamma-ray attenuating filler in silicone rubber matrices remains largely unexplored. From a radiation physics perspective, incorporating silica ash is expected to enhance attenuation by increasing composite and electron density, thereby improving Compton scattering at intermediate energies and contributing to photoelectric absorption at lower energies^7,12^.

In this context, the present study aims to develop silicone rubber composites reinforced with varying concentrations of silica ash as multifunctional gamma-ray shielding materials. The work focuses on experimentally determining the linear attenuation coefficient, mass attenuation coefficient, HVL, TVL, and MFP over a wide photon-energy range using standard gamma-ray sources, with validation against theoretical predictions from the NIST XCOM database^8^. In addition, the effects of silica ash loading on microstructure, mechanical performance, and thermal stability are investigated to establish comprehensive structure property shielding relationships^42^. this study seeks to advance eco-friendly and flexible alternatives to conventional heavy-metal-based gamma-ray shielding materials.

To the authors’ knowledge, this is the first systematic study to investigate waste-derived silica ash as a dual-function filler for simultaneous gamma-ray attenuation and mechanical reinforcement in silicone rubber matrices across a wide photon-energy range. The integration of experimental attenuation measurements, XCOM-based validation, lead-equivalent benchmarking, and comprehensive mechanical and thermal characterization provides new insight into the structure property shielding relationships of eco-friendly polymer shielding composites.

## Materials and composite preparation

### Silicone rubber matrix

The elastomeric phase utilized in this investigation was a commercial-grade RTV (Room-Temperature Vulcanization) polydimethylsiloxane (PDMS) system. The pristine matrix exhibited a nominal density of 1.1 g.cm^− 3^ and was selected for its high siloxane (Si-O-Si) backbone flexibility and structural stability.

The vulcanization process was initiated by the addition of a catalytic cross-linking agent (hardener) to ensure a consistent cross-linking density. The mixture was subjected to high-shear mechanical agitation to achieve macroscopic homogeneity, followed by a vacuum degassing phase (10 mbar) for 20 min to eliminate entrapped micro-voids a critical step to prevent photon leakage and ensure the validity of the narrow-beam geometry.

For the gamma-ray attenuation experiments, the composites were cast into precision-machined molds to produce cylindrical disk geometries. The final specimens were fabricated with a diameter of 5 cm and a calibrated thickness of 0.491 cm. The thickness was verified at multiple points using a digital micro-meter (precision ± 0.001 mm) to ensure a plane-parallel geometry, which is essential for minimizing uncertainty in the determination of the linear attenuation coefficient (µ) according to the Lambert-Beer law. The composites were cured under controlled ambient conditions (23 ± 2 °C) for 24 h. Complete cross-linking was confirmed by monitoring the physical state of the specimens; a “quiescent” state was reached when the material exhibited a tack-free surface and demonstrated full elastic recovery (zero permanent set) upon manual stretching to 100% elongation.

### Silica ash filler

#### Preparation of silica ash

The silica ash utilized in this study was sourced as a valorized siliceous byproduct from industrial smelting. To optimize the surface area and facilitate interfacial bonding within the elastomeric matrix, The silica ash was processed using a ball mill to ensure uniform particle size distribution and improved dispersion within the polymer matrix. The milling process was carried out using a ball-to-powder weight ratio (BPR) of 10:1, at a rotational speed of 500 rpm, for approximately 6 h under ambient conditions.

This top-down comminution process successfully shifted the particle size distribution (PSD) from the macro-scale to a hierarchical micro-nano regime, spanning from 5 μm down to approximately 500 nm. This polydisperse distribution was strategically intended to maximize the packing fraction within the polymer voids, thereby increasing the effective density for gamma-ray attenuation.

#### Fabrication of PDMS-silica ash composites

A series of binary composites were synthesized by integrating Silica Ash into a polydimethylsiloxane (PDMS) matrix at varying weight fractions (0–50 wt%: 0, 10, 20, 30, 40, 50 wt%). The fabrication followed a systematic high-shear integration protocol:

1. High-Shear Dispersion: The calculated mass of processed Silica Ash was introduced into the PDMS base in incremental stages. Subsequently, the milled silica ash was incorporated into the silicone rubber matrix and subjected to mechanical agitation using an industrial portable hand blender at a rotational speed of 300 rpm for 15 min to ensure homogeneous dispersion of the filler. This high-shear environment was critical to overcome the van der Waals cohesive forces between sub-micron Silica Ash particles, ensuring a homogeneous spatial distribution.
2. Degassing: To eliminate entrapped air and prevent the formation of structural micro-voids that could act as leakage paths for ionizing radiation, the mixtures were vacuum-degassed at a pressure of 10 mbar for 20 min prior to curing.
3. Curing/Vulcanization: The curing agent was added to the silicone rubber base at a stoichiometric ratio of 10:1 by weight (base: curing agent). The mixing process was carefully controlled (1–2 min) to prevent premature crosslinking, ensuring uniform blending while maintaining the elastomeric behavior of the composites. Room-Temperature Vulcanization (RTV) was maintained for 24 h under controlled environmental conditions (25 °C) to ensure consistent cross-linking density across all samples.

### Characterization of silica ash

#### Scanning electron microscopy (SEM)

The surface morphology and particle size distribution of the silica ash were investigated using a field-emission scanning electron microscope (FESEM, JEOL JSM-7900 F, JEOL Ltd., Japan). Prior to imaging, the silica ash powder was sputter-coated with a thin platinum (Pt) layer to enhance surface conductivity and minimize charging effects during electron-beam exposure. SEM micrographs were acquired under high-vacuum conditions using secondary electron (SE) imaging mode at an accelerating voltage of 30 kV.

#### Energy-dispersive X-ray spectroscopy (EDX)

The elemental composition of the silica ash was analyzed using an energy-dispersive X-ray spectroscopy (EDX) system integrated with the JEOL JSM-7900 F FESEM. EDX spectra were collected from multiple selected regions to ensure representative compositional analysis. Quantitative elemental analysis was performed using the Smart Quant software.

### Characterization of silicone rubber matrix

#### X-ray fluorescence (XRF) analysis

The elemental composition of the pristine silicone rubber matrix (0 wt% silica ash) was determined using X-ray fluorescence (XRF) analysis. Measurements were performed using an Oxford Instruments X-MET7000 series XRF spectrometer (XMDS 2726, serial no. 760073) operated at a maximum excitation voltage of 45 kV and a tube current of 50 µA. The measurements were conducted in bulk mode, ensuring representative analysis of the silicone rubber matrix.

#### Scanning electron microscopy (SEM)

The microstructural features of the silicone rubber/silica ash composites were examined using a field-emission scanning electron microscope (FESEM, JEOL JSM-7900 F, JEOL Ltd., Japan) operated at an accelerating voltage of 30 kV. SEM analysis was performed to qualitatively assess filler dispersion, filler-matrix interfacial bonding, and the presence of particle agglomeration within the polymer matrix.

#### X-ray fluorescence (XRF) analysis

The elemental composition of the silicone rubber composites was analyzed using X-ray fluorescence (XRF) with an Oxford Instruments X-MET7000 series spectrometer, operated in bulk measurement mode under the same conditions described for the pristine silicone rubber. XRF measurements were carried out for all filler concentrations to verify compositional consistency and the progressive incorporation of silica ash.

#### Density of silicone rubber composites

The densities of the prepared samples were measured using a hybrid automatic magnetic levitation system, enabling high-accuracy, non-contact density determination of solid materials. The measured densities were 1.12, 1.14, 1.16, 1.18, and 1.20 g·cm⁻³ for silica ash loadings of 10, 20, 30, 40, and 50 wt%, respectively. The monotonic increase in density reflects the higher intrinsic density of the silica ash compared to the silicone rubber matrix. The measured density values were incorporated into the calculation of mass attenuation coefficients and related shielding parameters.

#### Thermal analysis

**Fig. 1:**
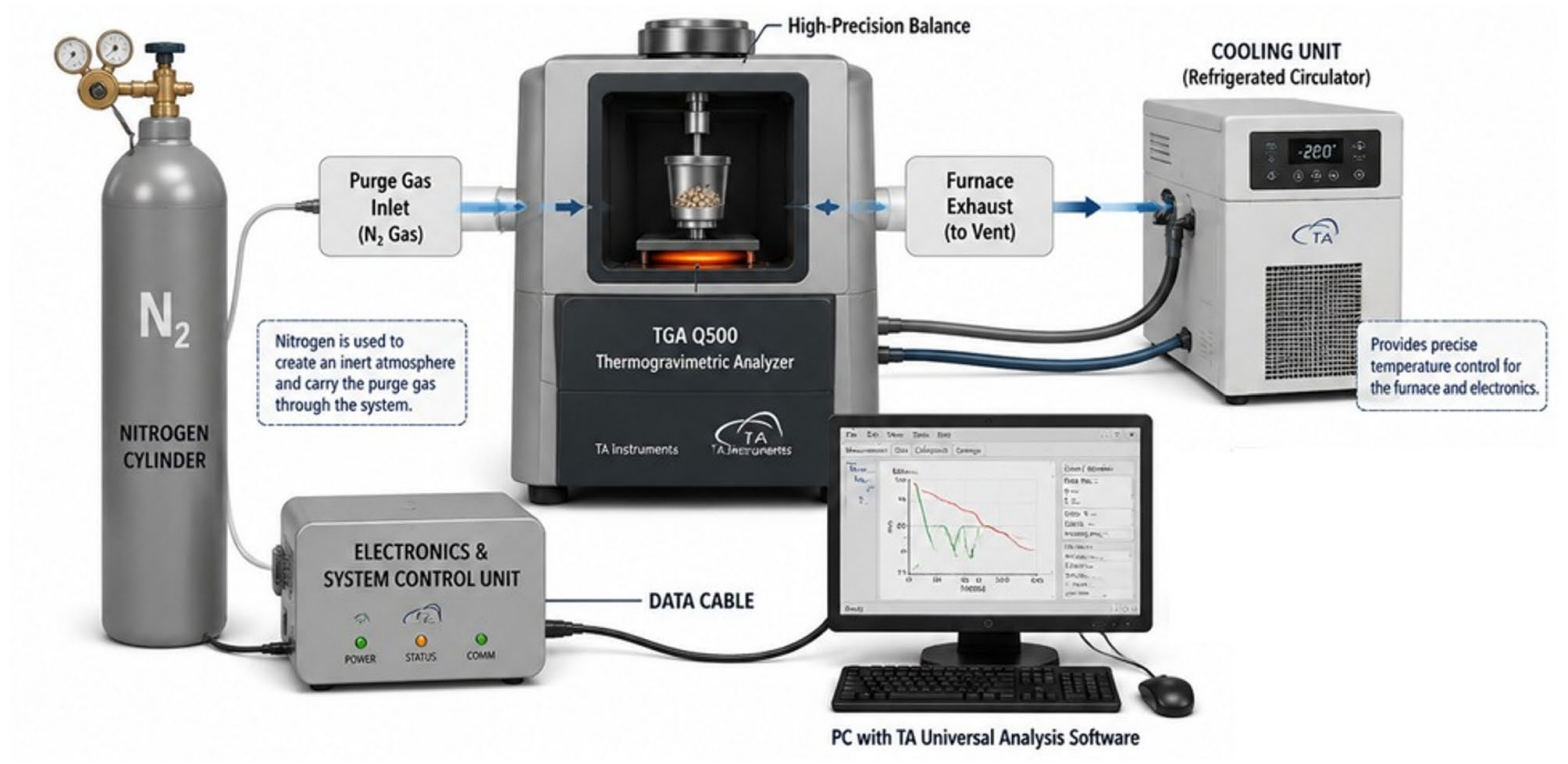
Schematic representation of the thermogravimetric analysis (TGA) experimental setup created by the authors using nanobanana.

The thermal stability and degradation behavior of the silicone rubber and silica-ash-filled silicone rubber composites were investigated using thermogravimetric analysis (TGA). Measurements were carried out using a TGA Q500 thermal analyzer (TA Instruments, USA) under an N2 atmosphere, The TGA instrument used in this study is shown in Fig. 1.

Approximately 19 mg of each sample was placed in a platinum crucible and heated from 25 °C to 900 °C at a constant heating rate of 10 °C·min⁻¹, The nitrogen gas flow rate was maintained at 60 mL.min^-1^ throughout the analysis. The mass loss of the samples was continuously recorded as a function of temperature. All measurements were performed under identical experimental conditions to ensure reliable comparison between the pristine silicone rubber and the silica-ash-filled composites.

### Mechanical testing

The mechanical properties of the silica ash-reinforced PDMS composites were evaluated using uniaxial Tensile specimens were prepared as rectangular strips with dimensions of 45 mm (length) × 6 mm (width) × 4.15 mm (thickness).

The tensile testing was conducted in accordance with the principles of ASTM D412 (Standard Test Methods for Vulcanized Rubber and Thermoplastic Elastomers-Tension). A constant crosshead speed of [130 mm.min^-1^] was maintained to ensure consistent strain rates across all compositions.

Tensile tests were conducted using a universal testing machine under ambient laboratory conditions (23 ± 2 °C). The tests were performed at a constant crosshead speed of 130 mm.min^-1^, which was maintained consistently throughout the measurements. The applied load and corresponding displacement were continuously recorded until specimen failure.

Engineering stress-strain curves were obtained by normalizing the applied force to the initial cross-sectional area of the specimens and the elongation to the original gauge length. The tensile strength was defined as the maximum stress prior to fracture, while the strain at rupture was determined from the elongation at break. The Young’s modulus was calculated from the slope of the initial quasi-linear elastic region of the engineering stress-strain curve, and the toughness was evaluated as the area under the stress-strain curve up to the point of fracture. All mechanical properties were performed using three independent specimens for each composition (*n* = 3), and the reported values represent the mean ± standard deviation.

### Gamma-ray spectroscopy setup

Gamma-ray attenuation measurements were performed using a set of standard radioactive point sources, including ²⁴¹Am, ¹³³Ba, ¹³⁷Cs, ⁶⁰Co, and ¹⁵²Eu, covering a wide photon-energy range from 59.53 keV to 1408.01 keV^43^. The photon energies and corresponding half-life times of the employed sources are summarized in Table 1. These sources provide a representative energy spectrum suitable for investigating different photon-matter interaction mechanisms, including photoelectric absorption and Compton scattering.

All radiation attenuation parameters, were performed using three independent replicates for each composition (*n* = 3), and the reported values represent the mean ± standard deviation.

All measurements were carried out at the Radiation Physics Laboratory, Alexandria University, Egypt, using a Canberra Type 802 NaI(Tl) scintillation detector^44^. The detector consists of a cylindrical NaI(Tl) crystal optically coupled to a photomultiplier tube through a 14-pin connector. The crystal dimensions are 76.2 mm in height and 38.1 mm in radius, providing high intrinsic detection efficiency for gamma-ray measurements^45^. The detector exhibits an energy resolution of approximately 8.5% at 661 keV, which is characteristic of NaI(Tl) scintillation systems and suitable for attenuation studies based on full-energy photopeak analysis^46^.

**Fig. 2:**
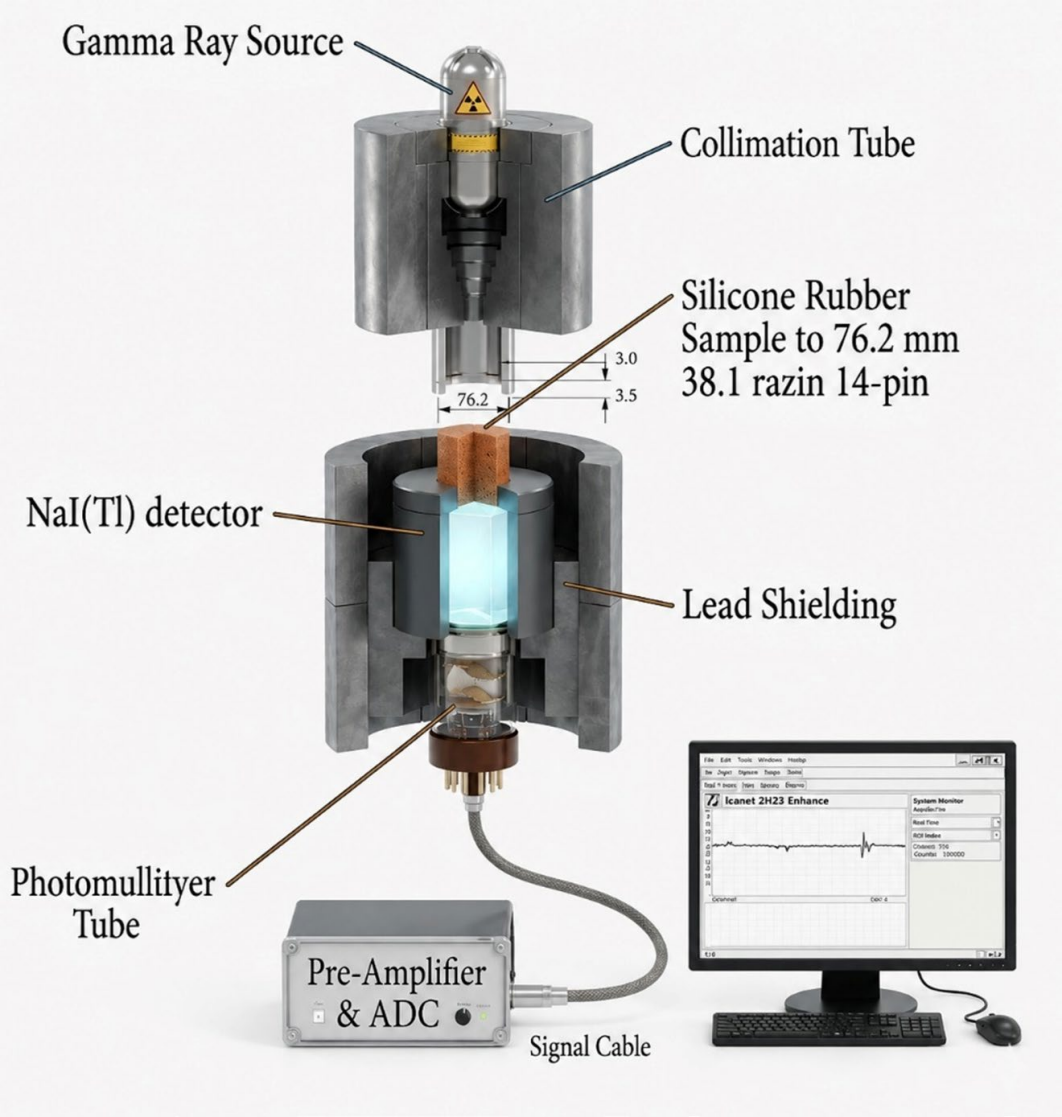
Schematic representation of the experimental setup for gamma-ray shielding measurements created by the authors using nanobanana.

During measurements, gamma photons transmitted through the composite samples interacted with the NaI(Tl) crystal, producing scintillation light proportional to the deposited photon energy. The resulting electrical pulses from the photomultiplier tube were processed and recorded as energy spectra using Genie 2000 software^47^. Each spectrum was acquired for a sufficiently long counting time to ensure reliable statistics, with the relative statistical uncertainty maintained below 1% for all measured photo peaks, The gamma-ray attenuation measurements were conducted using the experimental setup shown in Fig. 2.

The net photopeak areas corresponding to each gamma-ray energy were extracted after background subtraction and used to calculate the transmitted photon intensities. These values were subsequently employed to determine the linear attenuation coefficient and related shielding parameters using the Beer-Lambert law. To validate the experimental methodology, the measured attenuation coefficients were systematically compared with theoretical values obtained from the NIST XCOM database, confirming the reliability and accuracy of the experimental setup.

**Table 1:** Photon energies and half-life times of the radioactive sources used in the gamma-ray attenuation measurements.

Radioactive source	Photon energy (keV)	Half-life (days)
²⁴¹Am	59.5	157,680
¹³³Ba	80.99	3,847.91
¹³⁷Cs	661.66	11,004.98
⁶⁰Co	1173	1,925.31
⁶⁰Co	1332.5	1,925.31

## Theoretical background

### Radiation shielding parameters

The interaction probability of gamma photons with matter per unit path length is described by the linear attenuation coefficient (µ), expressed in cm⁻¹. Experimentally, µ is determined using the Beer-Lambert law, which relates the transmitted photon intensity to the absorber thickness:

1 $$\:I={I}_{o}\:{e}^{-\mu\:x}$$

where *I*_*o*_ and *I* represent the incident and transmitted photon intensities, respectively, and x is the thickness of the absorbing material. Rearranging the equation yields the linear attenuation coefficient:

2 $$\:{\upmu\:}=\:\frac{1}{x}\:\mathrm{ln}\frac{{I}_{o}}{I}$$

To account for density variations among different composite samples, the mass attenuation coefficient (MAC) in (cm²·g⁻¹), was calculated by normalizing the linear attenuation coefficient to the sample density ρ:

3 $$\:{{\upmu\:}}_{m}=\:\frac{{\upmu\:}\:}{{\uprho\:}}$$

The experimentally obtained MAC values were compared with theoretical values calculated using the NIST XCOM database, which provides photon interaction cross sections based on elemental composition and photon energy.

Several additional radiation shielding parameters were derived from the linear attenuation coefficient to assess shielding effectiveness:

Half-value layer (HVL), defined as the material thickness required to reduce the photon intensity to 50% of its original value:

4 $$\:{\mathrm{HVL}}=\:\frac{\mathrm{ln}2}{{\upmu\:}}$$

Tenth-value layer (TVL), corresponding to a 90% reduction in photon intensity:

5 $$\:{\mathrm{TVL}}=\:\frac{\mathrm{ln}10}{{\upmu\:}}$$

Mean free path (MFP), representing the average distance traveled by a photon between successive interactions:

6 $$\:{ \mathrm{MFP}}=\:\frac{1}{{\upmu\:}}$$

To evaluate the accuracy of the experimental results, the percentage deviation between experimental and theoretical (XCOM) attenuation coefficients was calculated using:

7 $$\:{\Delta}_{\mathrm{error}}\:=\frac{{\:{\upmu\:}}_{\mathrm{xcom}}\:-{\mu\:}_{\mathrm{Exp}}\:}{{\mu\:}_{\mathrm{Exp}}}\:\times\:100\:$$

This comparison provides a quantitative measure of agreement between experimental measurements and theoretical predictions.

### Mechanical parameters

The mechanical performance of the silicone rubber and silica-ash-reinforced composites was evaluated using tensile testing. The engineering stress (σ) and engineering strain (ε) were determined from the applied load and deformation, respectively.

The Young’s modulus (E), which quantifies the elastic stiffness of the material, was calculated from the slope of the linear elastic region of the stress-strain curve:

8 $$\:E=\:\frac{{\upsigma\:}}{{\upepsilon\:}}$$

9 $$\:{\upepsilon\:}=\frac{\mathrm{elongation}\:\%}{100}$$

10 $$\:{\upsigma\:}=\frac{mg}{Ao}$$

Toughness, quantified as the area under the stress-strain curve, it can be expressed as:

11 $$\:{U}_{t}=\:{{\int_{\:0}}^{\:\upepsilon}}\sigma\:\left(\upepsilon\:\right)d\upepsilon\:$$

where $\:{U}_{t}$ is the toughness (energy per unit volume, typically in MJ·m⁻³).

A numerical integration method was employed for accurate quantification. The trapezoidal rule was applied to the discrete stress-strain data pairs (${\upepsilon}_{i}$,$\:\:{\sigma\:}_{i}$) obtained from the tensile test, providing a robust approximation of the integral:

12 $$\:{U}_{t}=\sum\:_{i}^{n-1}\frac{\left({\sigma\:}_{i}+{\sigma\:}_{i+1}\right)}{2}.({\upepsilon}_{i+1}-{\upepsilon}_{i})$$

These mechanical parameters were used to assess the influence of silica ash loading on stiffness, load-bearing capability, and energy storage capacity of the composites, and to correlate mechanical reinforcement with radiation shielding performance.

## Results and discussion

### Characterization of silica ash (SEM and EDX)

#### Morphology (SEM)

**Fig. 3:**
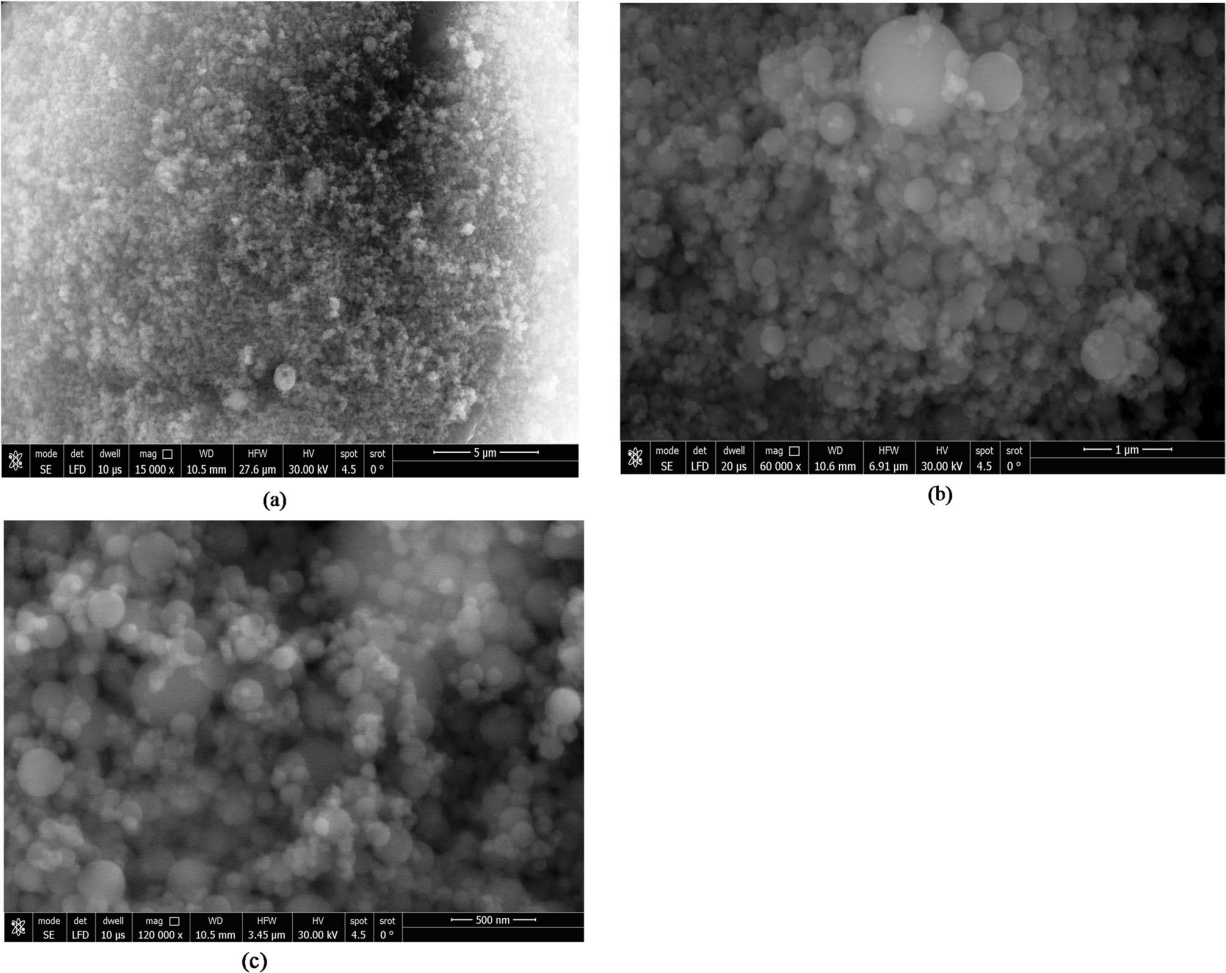
SEM micrographs of silica ash at different magnifications showing (**a**) overall particle morphology at 5 μm, (**b**) particle size distribution at 1 μm, and (**c**) nanoscale features at 500 nm.

SEM analysis (Fig. 3) reveals a heterogeneous, polydisperse morphology stemming from the stochastic ball-milling process. The particles exhibit a hierarchical distribution, ranging from the micro-meter scale down to sub-micron dimensions = 500 nm.

This multiscale architecture is strategically advantageous: the micro-particles provide structural rigidity, while the nanometric fraction increases the specific surface area, enhancing interfacial kinetics within the polymer matrix. High-magnification imaging confirms an asperity-rich surface texture, which facilitates mechanical interlocking and promotes robust filler-matrix adhesion. Despite localized van der Waals-driven agglomeration typical of high-surface-energy siliceous fillers the rough morphology ensures high reinforcing efficiency in the silicone rubber composite, The surface morphology of the silica ash particles at different magnifications is presented in Fig. 3.

#### Elemental composition (EDX)

**Fig. 4:**
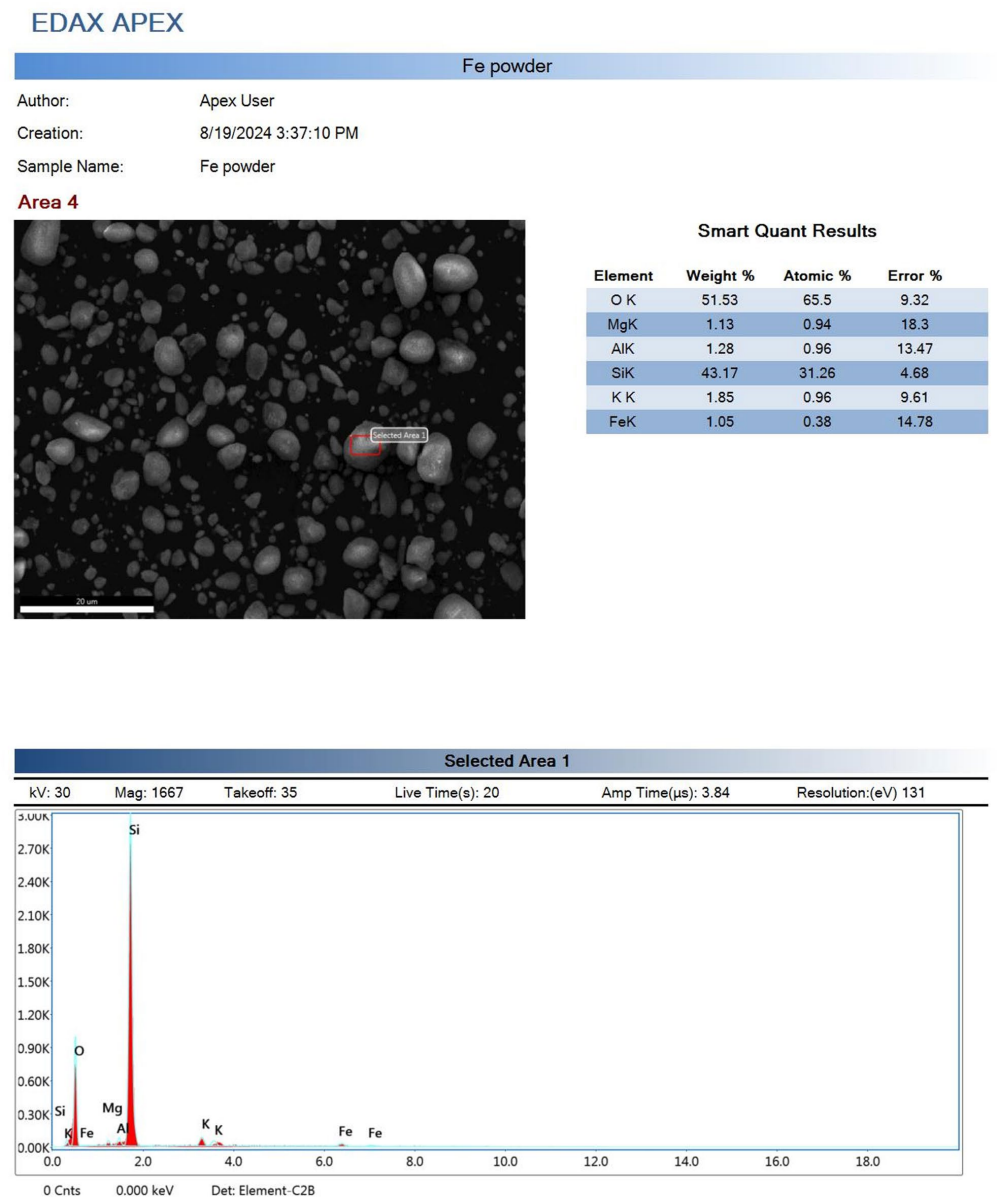
EDX spectrum of silica ash showing the dominance of Si and O, confirming its SiO₂-rich composition, and corresponding elemental composition table.

EDX spectroscopy (Fig. 4) confirms a predominantly siliceous composition, with silicon (43.71 wt%) and oxygen (51.53 wt%) peaks validating SiO_2_**-**rich framework.

Minor industrial constituents, including Mg, Al, K, and Fe, were detected in trace quantities (< 2 wt%). These secondary elements are strategically significant; specifically, the presence of higher-Z elements like K and Fe modulates the effective atomic number Z_eff_ and electron density of the filler. This alteration enhances the probability of gamma-ray interactions via Compton scattering, particularly in the intermediate energy regime.

Consequently, the synergistic SEM/EDX characterization establishes the processed silica ash as a viable bifunctional filler, providing both mechanical reinforcement and enhanced radiation attenuation within the silicone rubber matrix.

### Morphological and chemical analysis of silicone rubber (SEM and XRF)

#### Chemical/bulk analysis (XRF)

XRF spectroscopy (Table 2) validates the siloxane architecture of the pristine matrix, which is dominated by SiO_2_ (93.14 wt%) and minor residual catalysts (e.g., Al_2_O_3_, Fe_2_O_3_, ZnO) From a radiation-physics perspective, this low-Z primary composition establishes a baseline of limited intrinsic gamma-ray attenuation. The incorporation of 30 wt% silica ash (Table 3) induces a strategic compositional shift, significantly elevating the concentration of higher Z constituents. From a radiation-physics perspective, this modification is particularly significant in the low-energy regime, where the photoelectric effect is the primary attenuation driver.

Since the photoelectric cross-section (τ) scales with the atomic number as Z^4–5^, the trace presence of elements such as Fe (Z = 26), K (Z = 19), and Ag (Z = 47) disproportionately enhances the interaction probability compared to the low-Z siloxane matrix. Furthermore, the increased electron density associated with the ash filler optimizes photon attenuation via Compton scattering in the intermediate energy region. Consequently, the waste-derived silica ash acts as a dual-spectrum enhancer, improving shielding performance across both photoelectric-dominated and scattering-dominated regimes. These XRF-derived elemental mass fractions were utilized as primary input parameters for NIST XCOM simulations, ensuring a high-fidelity correlation between theoretical predictions and experimental mass attenuation coefficients, complete compositional data for the 10–50 wt% range are detailed in the Supplementary Information.

**Table 2:** XRF quantitative elemental and oxide composition of pristine silicone rubber (0 wt% silica ash) measured in bulk mode.

Ag	Al	Ca	Cr	Fe	Mo	Nb
0.0800	0.28	0.40	0.03	0.08	0.02	0.02
Ag2O	Al2O3	CaO	Cr2O3	FeO	MoO3	Nb2O5
0.09	0.53	0.56	0.04	0.10	0.03	0.03
				Fe2O3		
				0.11		
P	S	Si	Sn	Ti	Zn	Zr
0.12	1.10	43.54	0.15	0.12	0.0400	0.0100
P2O5	SO3	SiO2	SnO2	TiO2	ZnO	ZrO2
0.27	2.75	93.14	0.19	0.20	0.05	0.01
			SnO			
			0.17			

**Table 3:** XRF elemental and oxide composition of the silicone rubber composite containing 30 wt% silica ash, used to determine effective atomic number and electron density for gamma-ray attenuation calculations.

Ag	Al	Ca	Cl	Co	Cu	Fe	K	Mn
0.0200	0.53	0.54	1.65	0.01	0.01	0.63	0.34	0.04
Ag2O	Al2O3	CaO	Cl	CoO	CuO	FeO	K2O	MnO
0.02	1.00	0.76	1.65	0.01	0.01	0.81	0.41	0.05
						Fe2O3		
						0.90		
Mo	Nb	Ni	P	S	Si	Sn	Ta	Ti
0.01	0.01	0.01	0.19	1.18	41.27	0.17	0.01	0.13
MoO3	Nb2O5	NiO	P2O5	SO3	SiO2	SnO2	Ta2O5	TiO2
0.02	0.01	0.01	0.44	2.95	88.28	0.22	0.01	0.22
						SnO		
						0.19		
W	Zn	Zr						
0.0200	0.0200	0.0300						
WO3	ZnO	ZrO2						
0.03	0.02	0.04						

#### Surface morphology (SEM)

##### Pure silicone rubber (Baseline)

**Fig. 5:**
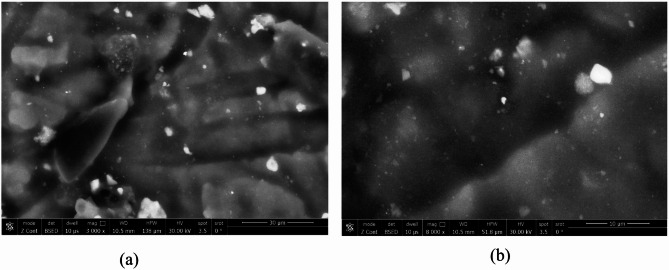
SEM analysis of the pure silicone rubber, SEM micrographs of the pure silicone rubber matrix (0 wt% silica ash) recorded in BSE mode at different magnifications: (**a**) 3000×, and (**b**) 8000**×**.

SEM analysis of the pure silicone rubber (Fig. 5) reveals a monolithic, single-phase polymer matrix with uniform contrast across all magnifications. The surface is featureless and isotropic, devoid of voids, micro-fractures, or phase-separated domains. This quiescent morphology confirms the integrity of the cured elastomer and establishes a baseline reference, accounting for the matrix’s low intrinsic gamma-ray attenuation efficiency and high chain mobility.

##### 15 wt% composite: optimized dispersion

**Fig. 6:**
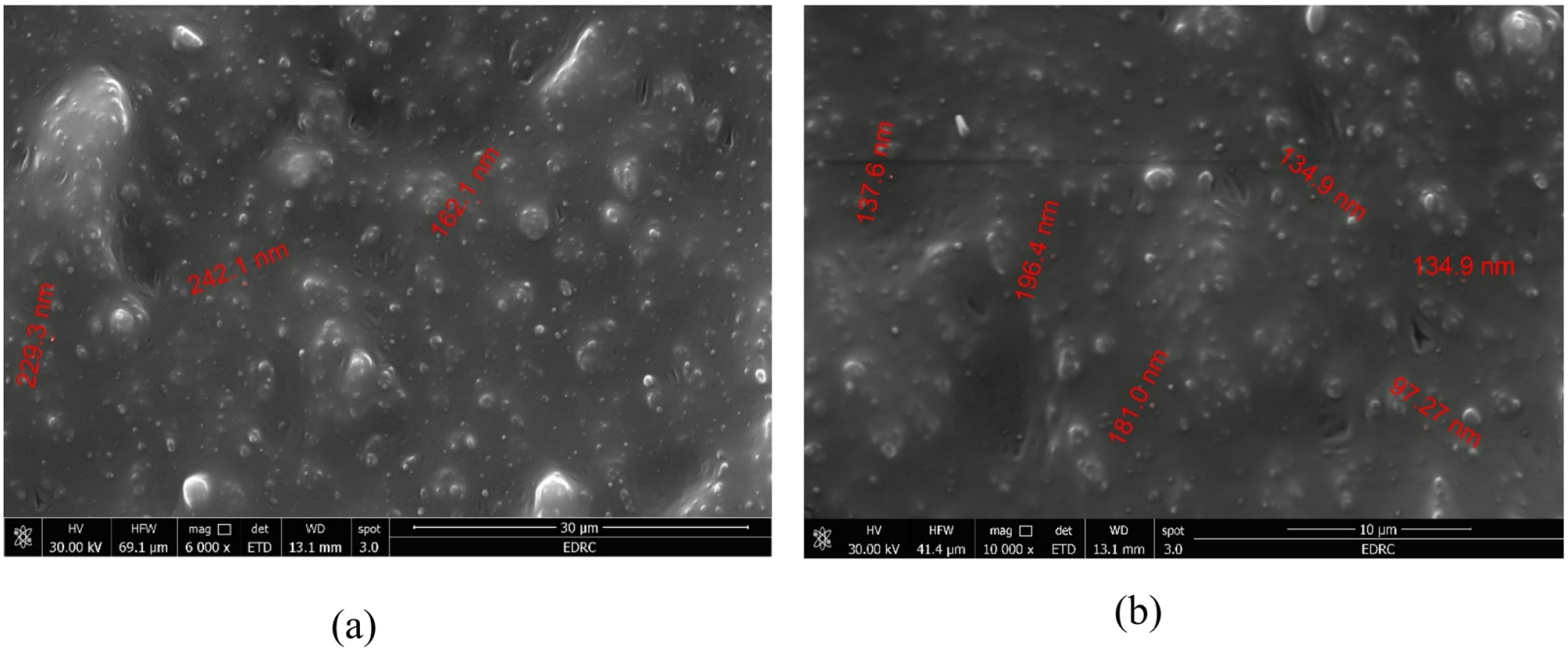
FESEM micrographs of the 15 wt% silica ash/siloxane composite at multiple scales: (**a**) Low-magnification view (6000**×**) demonstrating a relatively uniform spatial distribution of the filler phase within the polymer matrix; (**b**) High-magnification micrograph (10000**×**) highlighting the presence of discrete nanoparticles and minor, low-order clusters. Red annotations indicate primary particle diameters in the range of 100–250 nm. The lack of large-scale, irregular agglomerates confirms the efficacy of the dispersion process at this loading level.

The morphology of the 15 wt% silica ash composite (Fig. 6) demonstrates a relatively uniform filler distribution within the siloxane network. High-magnification micrographs (6000**×** to 1000**×**) identify the silica ash primarily as discrete primary nanoparticles and localized, low-order clusters. The red annotations in Fig. 6b confirm particle sizes within the nano-regime (~ 100–250 nm), suggesting that the filler remains in the pre-percolation regime.

Critically, the interfacial transition zone (ITZ) appears seamless; the matrix effectively “wets” the filler surfaces without observable debonding or interfacial voids. This robust coupling facilitates efficient stress transfer, directly correlating with the high ductility and toughness observed in the mechanical testing. This configuration represents a microstructural optimum, providing stable, isotropic gamma-ray attenuation without compromising the elastomer’s flexibility.

##### 50 wt% composite: agglomeration-induced embrittlement

**Fig. 7:**
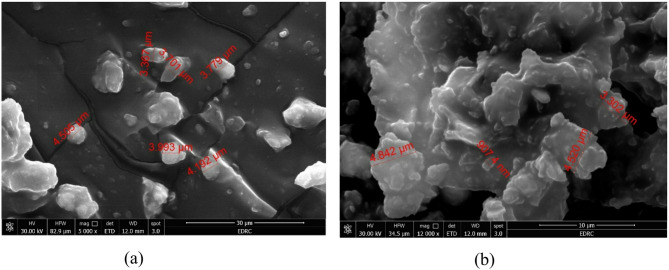
FESEM micrographs of the 50 wt% silica ash/siloxane composite illustrating high-loading morphology: (**a**) Low-magnification micrograph (5000**×**) revealing severe, large-scale filler agglomeration distributed throughout the matrix; (**b**) High-magnification view (12000**×**) identifying secondary clusters with dimensions ranging from 3 to 5 μm. These massive agglomerates act as primary stress concentrators, initiating the observed quasi-brittle fracture planes. The transition from minor localized aggregates (at 15 wt%) to these micrometer-scale clusters confirms the mechanism of agglomeration-induced embrittlement and the subsequent loss of material extensibility.

As the filler concentration increases to 50 wt% (Fig. 7), a profound morphological shift occurs. The micrographs reveal severe, large-scale filler agglomeration, with secondary clusters reaching micrometer-scale dimensions (3–5 μm), as shown in Fig. 7a and b.

Unlike the 15 wt% sample, the 50 wt% composite exhibits a jagged, quasi-brittle fracture surface. These massive agglomerates act as high-intensity stress concentrators that disrupt the continuity of the siloxane network and initiate premature micro-cracking. This visual evidence confirms that the reduction in elongation and the shift toward brittle fracture behavior are physically driven by agglomeration-induced embrittlement, where the oversized clusters overcome the matrix’s ability to undergo ductile deformation.

### Mechanical characterization and interfacial reinforcement dynamics

#### Constitutive stress-strain evolution

The tensile response of the silica ash/silicone rubber (SR) composites exhibits the non-linear hyper elasticity characteristic of cross-linked siloxane networks. As summarized in Table 4, the transition from the pristine matrix to highly loaded composites (40 wt%) reveals a fundamental shift in deformation physics. The pristine SR is governed by entropic elasticity, where tensile energy is dissipated through macromolecular chain uncoiling, resulting in a high failure strain (ε_r_) of 508% and a low tensile strength (σ_u_) of 0.221 MPa.

Upon the introduction of silica ash, the constitutive curves demonstrate a progressive increase in the initial slope, signaling the onset of filler-induced kinematic constraints. This mechanical evolution is driven by the high surface-area-to-volume ratio of the ash particles, which act as physical cross-links that restrict the segmental mobility of the polydimethylsiloxane (PDMS) chains.

**Table 4:** Mechanical properties of silica ash-reinforced PDMS composites (mean ± standard deviation, *n* = 3).

Ash content %	Tensile strength (MPa)	Young’s modulus E (MPa)	Strain at rupture	Toughness (MJ·m⁻³)
0%	0.2210 ± 0.0078	0.0260 ± 0.0013	5.0800 ± 0.2030	0.5050 ± 0.0178
10%	0.2930 ± 0.0147	0.0388 ± 0.0015	4.7000 ± 0.2179	0.5680 ± 0.0302
15%	0.3570 ± 0.0090	0.0990 ± 0.0053	2.9600 ± 0.1082	0.5910 ± 0.0127
20%	0.2610 ± 0.0113	0.1030 ± 0.0044	2.7600 ± 0.1277	0.3785 ± 0.0180
25%	0.2590 ± 0.0135	0.1350 ± 0.0062	2.3200 ± 0.1253	0.3220 ± 0.0160
30%	0.2070 ± 0.0115	0.1430 ± 0.0151	1.6800 ± 0.1114	0.1680 ± 0.0106
40%	0.1960 ± 0.0125	0.1570 ± 0.0137	1.4000 ± 0.1000	0.1350 ± 0.0140

#### Elastic stiffening and hydrodynamic reinforcement

The Young’s modulus (E) exhibits a robust six-fold enhancement, rising monotonically from 0.026 MPa in the pristine matrix to 0.157 MPa at 40 wt% loading. This stiffening can be attributed to the hydrodynamic and micromechanical reinforcement effect, where the rigid ash particles amplify the internal strain field of the compliant elastomeric matrix.

At low loadings (≤ 15 wt%), reinforcement is primarily interfacial, governed by the formation of an immobilized polymer layer (the “shell” effect) around individual ash particles. However, as loading reaches the percolation threshold (> 20 wt%), the modulus follows a near-linear trajectory, suggesting the formation of a filler-supported skeletal network. In this regime, the silica ash particles transition from isolated stress-concentrators to a continuous load-bearing phase, significantly elevating the composite’s structural rigidity. The pronounced increase in Young’s modulus can be further interpreted within the framework of classical micromechanical models such as Halpin-Tsai and Mori-Tanaka. These models describe the effective stiffness of particulate composites as a function of filler volume fraction, stiffness contrast, and load transfer efficiency at the interface. In the present system, the large modulus mismatch between the rigid silica ash particles and the compliant PDMS matrix leads to an efficient redistribution of applied stress toward the filler phase.

As the ash content increases, the probability of particle-particle interactions rises, promoting the formation of a semi-continuous load-bearing network. This transition enhances the effective stiffness beyond that expected from isolated particle reinforcement. Simultaneously, the confinement of polymer chains in the vicinity of the filler surface reduces segmental mobility, further contributing to stiffness enhancement. These combined effects explain the observed multi-fold increase in Young’s modulus with increasing filler loading.

#### Tensile strength optimization and fracture toughness

A critical non-monotonic trend is observed in the ultimate tensile strength and toughness, both of which peak at a 15 wt% threshold.

##### The reinforcement peak

At 15 wt%, the tensile strength reaches a maximum of 0.357 MPa 61% increase over the pristine matrix-while the toughness peaks at 0.591 MJ·m⁻³. This “sweet spot” represents the optimal balance of filler dispersion and interfacial adhesion. The rough surface morphology of the silica ash facilitates strong mechanical interlocking and potential hydrogen bonding between silanol groups and the SR backbone, maximizing energy dissipation before fracture.

##### Agglomeration induced failure

Beyond 15 wt%, both strength and toughness undergo a significant decline, with the 40 wt% sample falling to 0.196 MPa and 0.135 MJ·m⁻³, respectively. This degradation is a hallmark of filler agglomeration. In high-loading regimes, filler-filler interactions dominate over filler-matrix interactions, creating microstructural inhomogeneities that act as nucleation sites for crack propagation.

##### Ductile to brittle transition

The monotonic collapse of elongation at break from 508% to 140% confirms a transition from a ductile, chain-slippage failure mode to a brittle, filler-governed fracture. The rigid ash network prevents the macromolecular chains from undergoing the long-range orientation required for high strain, leading to premature catastrophic failure.

#### Macromolecular chain dynamics and ductility loss

The monotonic decline in strain at rupture (ε_r_) from 508% in the pristine matrix to 140% at 40 wt% loading reflects the restriction of macromolecular conformational entropy. Silica ash particles act as rigid steric hindrances, restricting polydimethylsiloxane (PDMS) chains through interfacial interactions and significantly reducing the free volume required for segmental mobility. At high mass fractions (> 20 wt%), the development of a percolated filler network induces strain localization at the matrix-filler interface, triggering a transition from hyper elastic uncoiling to quasi-brittle failure. Increasing ash (filler) content changes how the material breaks: it shifts from a stretchy, ductile silicone rubber to a brittle, less flexible composite. This happens because high concentrations of filler create stress points that cause the material to tear or fracture easily.

#### Toughness and energy dissipation optimization

Toughness ($\:{U}_{t}$), representing the volumetric energy absorption capacity, exhibits a non-monotonic “volcano-plot” trend, peaking at 0.591 MJ·m⁻³ at a 15 wt% ash loading. This 17% enhancement over the control sample (0.505 MJ·m⁻³) is driven by micromechanical dissipation mechanisms, such as microcrack deflection and filler debonding, which effectively dissipate stress within the toughening regime. Conversely, the sharp collapse to 0.135 MJ·m⁻³ at 40 wt% signals interfacial saturation and particle agglomeration. These clusters introduce macro-defects that bypass the matrix’s energy-absorption capabilities, identifying 15 wt% as the critical reinforcement threshold for durable shielding applications.

**Fig. 8:**
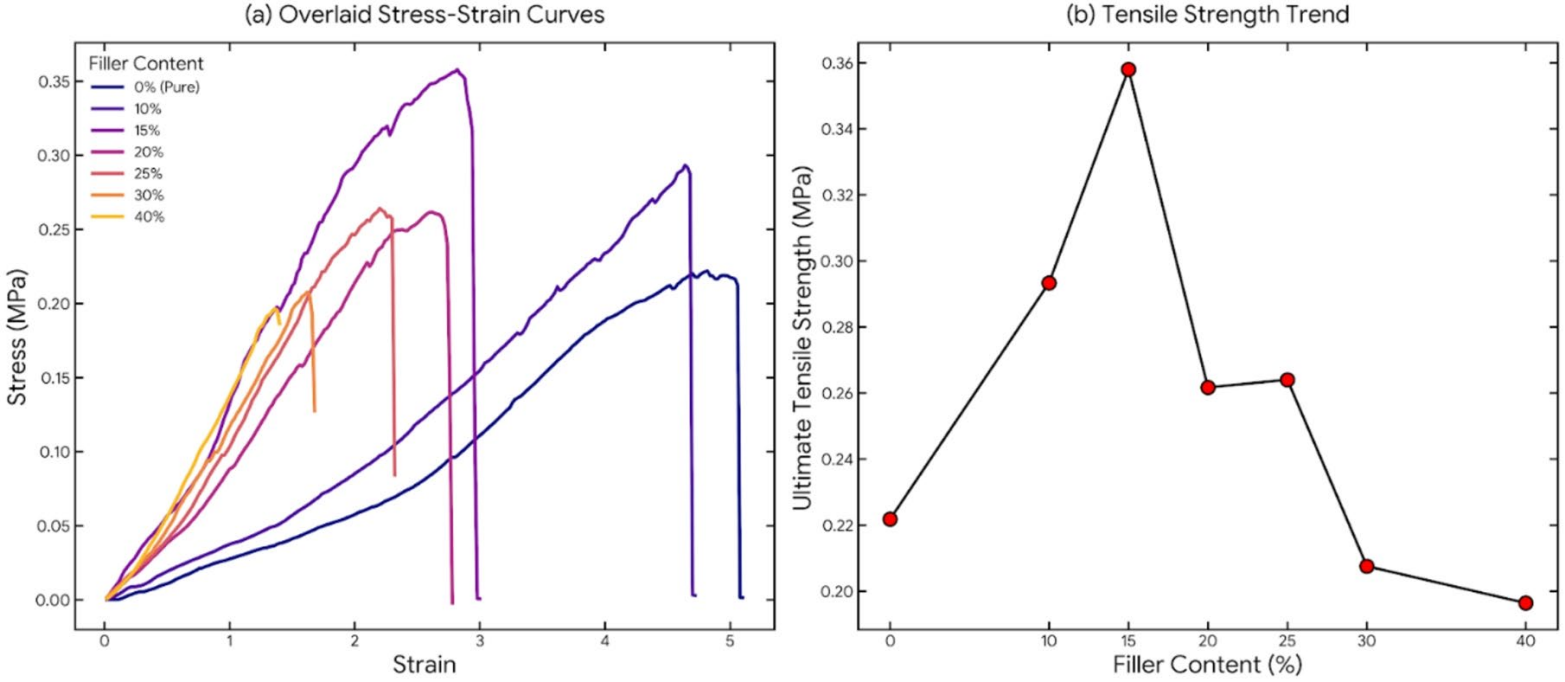
(**a**) Stress-strain curves of silica ash-reinforced PDMS composites with varying filler loadings (0 to 40 wt%), illustrating the evolution of mechanical behavior with increasing silica ash content. All curves are presented on identical axes to enable direct comparison of stiffness, elongation, and failure characteristics. (**b**) Variation of ultimate tensile strength as a function of filler content, highlighting the optimal mechanical performance at intermediate loadings and the subsequent decline at higher concentrations due to filler agglomeration effects.

The stress-strain behavior and tensile strength variation of the silica ash-reinforced PDMS composites are shown in Fig. 8. The curves reveal a progressive increase in stiffness (Young’s modulus) accompanied by a reduction in elongation at break with increasing silica ash content, indicating a transition from highly ductile elastomeric behavior toward a stiffer, filler-dominated mechanical response.

**Fig. 9:**
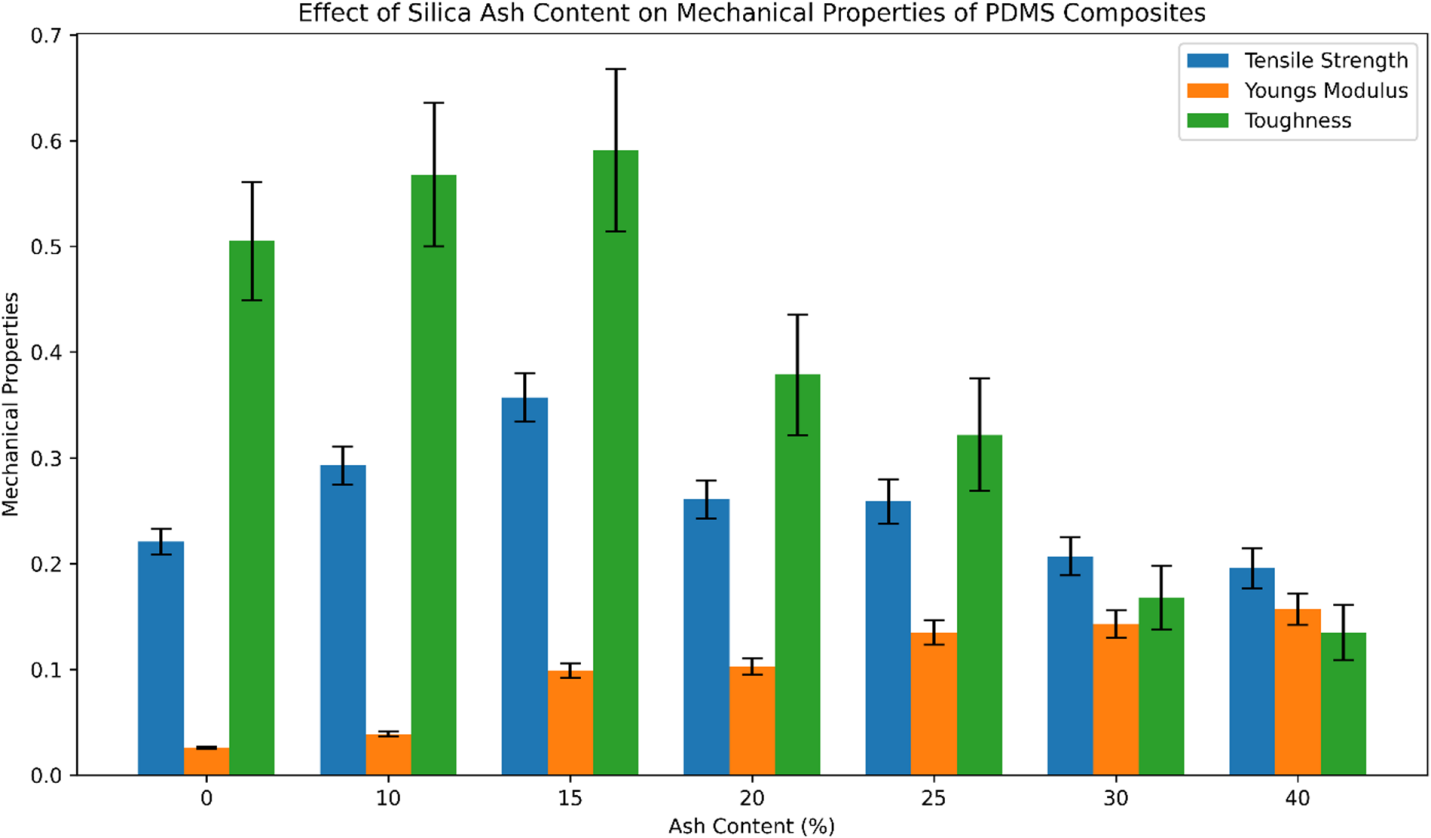
Effect of silica ash content on the mechanical properties of PDMS composites. The variation of tensile strength, Young’s modulus, and toughness is presented as a function of filler loading (0 to 40 wt%), mean ± standard deviation, *n* = 3. Error bars represent standard deviation (*n* = 3). The results demonstrate an optimal mechanical performance at intermediate filler content (≈ 15 wt%), followed by a decline in ductility and toughness at higher loadings due to filler agglomeration and reduced polymer chain mobility.

The effect of silica ash loading on the mechanical properties of the PDMS composites is illustrated in Fig. 9.

##### Statistical analysis of mechanical properties

To quantitatively assess the influence of silica ash content on the mechanical behavior, one-way analysis of variance (ANOVA) was performed. The results reveal a highly significant effect of filler loading on all measured properties, including tensile strength (F = 72.58, *p* < 0.001), Young’s modulus (F = 107.35, *p* < 0.001), strain at rupture (F = 272.20, *p* < 0.001), and toughness (F = 312.98, *p* < 0.001). These findings confirm that the observed variations are statistically meaningful and strongly governed by the ash content.

Notably, the higher F-values associated with strain and toughness indicate greater sensitivity of these properties to filler incorporation, reflecting pronounced structural changes within the composite system. The results further support the proposed reinforcement mechanism, where improved filler-matrix interaction enhances stiffness, while excessive filler loading leads to reduced ductility due to particle agglomeration and limited interfacial efficiency.

Detailed ANOVA results are provided in Table S2 (Supplementary Information).

#### Comparison with previously reported silicone rubber composites

A direct comparison with previously reported silica-filled silicone rubber composites shows good agreement with the present results. In the literature, the tensile strength increases from 0.21 MPa at 15 phr (≈ 13 wt%) to a maximum of 0.67 MPa at 100 phr (≈ 50 wt%**)**^48^, followed by a decline at higher filler loadings due to particle agglomeration. by comparison, the present study demonstrates an increase in tensile strength from 0.221 MPa (neat PDMS) to 0.357 MPa at 15 wt%, followed by a gradual decrease at higher filler content.

Importantly, at comparable filler loading (~ 15 wt%), the present composites exhibit a significantly higher tensile strength than previously reported systems (0.357 MPa vs. 0.21 MPa), indicating enhanced reinforcement efficiency. Although higher absolute tensile strength values are reported in the literature, these are achieved at substantially higher filler loadings.

**Fig. 10:**
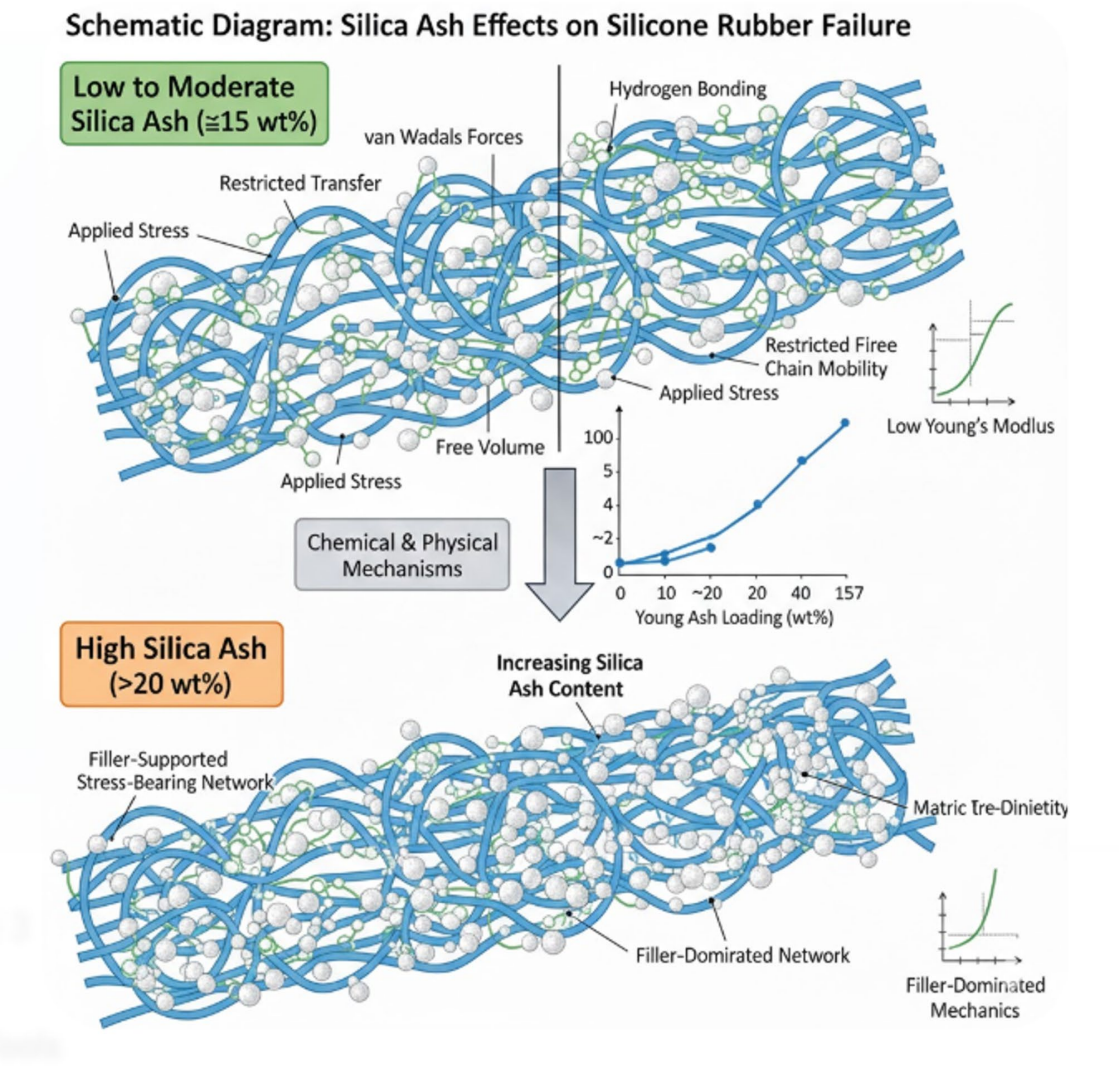
Schematic illustration of the load-transfer and reinforcement mechanisms governing the mechanical response of silica-ash/silicone rubber composites. (Top panel) At low-to-moderate loadings (≤ 15 wt%), uniformly dispersed silica ash particles form strong physicochemical interactions (e.g., hydrogen bonding, van der Waals forces) with the siloxane backbone. This interfacial coupling restricts macromolecular chain mobility, enhancing stress transfer and optimizing tensile strength and toughness. (Bottom panel) At high loadings (> 20 wt%), the system transitions to a filler-dominated mechanics regime. The formation of a rigid, percolated particle network significantly increases the Young’s modulus (central inset) but leads to particle agglomeration. These clusters act as stress concentration sites, restricting chain dynamics and promoting a transition to brittle failure.

From a practical perspective, achieving improved mechanical performance at relatively lower filler content is advantageous, as it helps preserve elastomeric flexibility, reduce material density, and maintain processability. The observed reduction in mechanical performance at higher filler concentrations in both studies is attributed to filler agglomeration and reduced interfacial efficiency, The reinforcement mechanism is illustrated in Fig. 10.

### Thermal stability and degradation kinetics

#### TGA analysis: the stability residue paradox

**Fig. 11:**
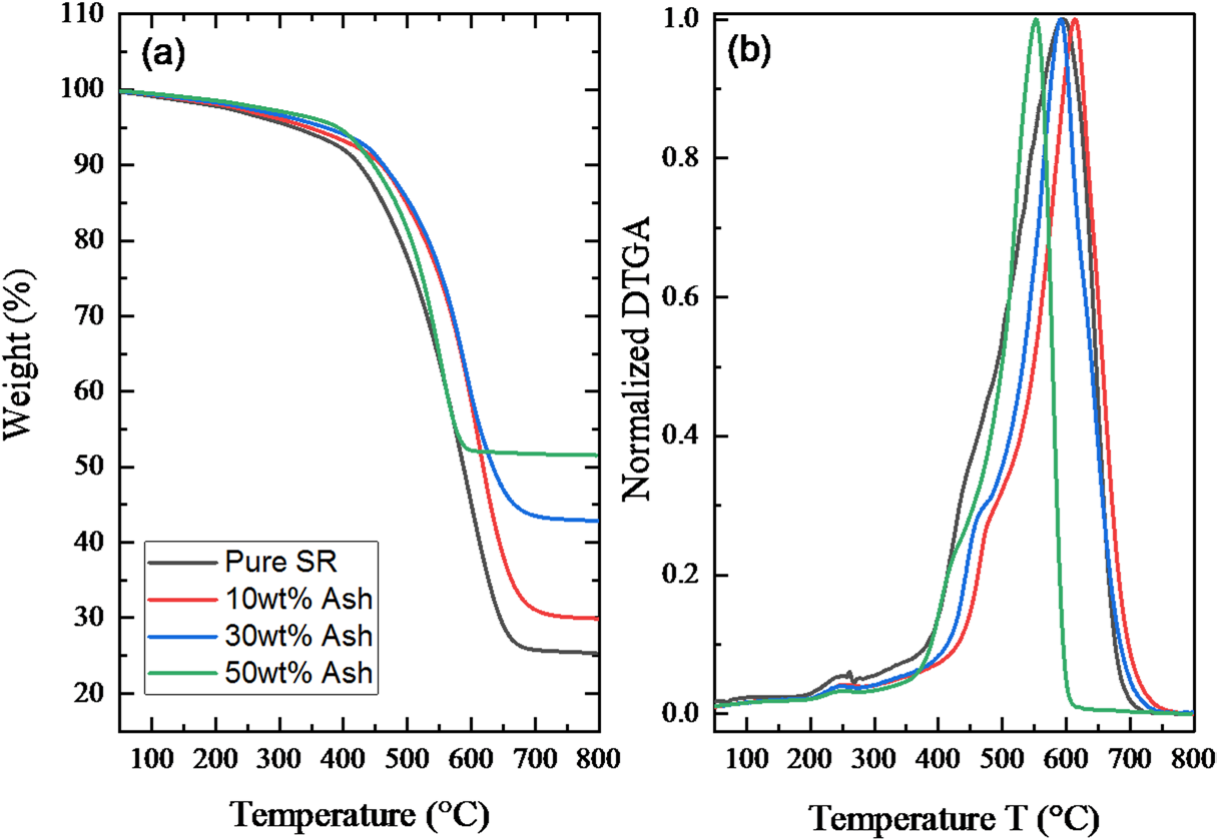
Thermogravimetric analysis of silicone rubber (SR) composites reinforced with silica ash at different loadings (0, 10, 30, and 50 wt%). (**a**) TGA curves showing mass loss as a function of temperature, highlighting the progressive enhancement in thermal stability and char yield with increasing silica ash content. (**b**) Corresponding normalized DTGA profiles illustrating the shift in maximum degradation temperature and the modification of decomposition kinetics induced by filler incorporation.

Thermogravimetric analysis (TGA) profiles reveal a complex relationship between silica ash loading and the thermo-oxidative stability of the silicone rubber (SR) matrix. While the final inorganic residue at 800 °C increases monotonically with filler loading rising from ≈ 25% in pristine SR to ≈ 52% in the 50 wt% composite the onset of degradation (T_onset_) follows a non-linear trend. At low loading (10 wt%), a subtle increase in thermal stability is observed, likely due to the physical barrier effect of well-dispersed ash particles, which hinders the diffusion of volatile cyclic siloxanes as in Fig. 11. However, at higher loadings (30–50 wt%), T _onset_ shifts significantly toward lower temperatures. The non-monotonic thermal behavior arises from a competition between stabilization and catalytic degradation mechanisms governed by filler loading. At low ash content (≈ 10 wt%), the dispersed silica-rich particles act primarily as a physical barrier, restricting chain mobility and suppressing the diffusion of volatile cyclic siloxane species. This results in a delayed onset of thermal degradation and a shift of the maximum degradation temperature (T-max) toward higher values.

At higher loadings (30–50 wt%), this stabilizing effect is progressively offset by the increasing density of surface-active sites associated with the ash particles. In particular, surface silanol (-OH) groups and metal oxide constituents may introduce localized acid-base interactions with the PDMS backbone, which can promote chain scission reactions. As a consequence, the degradation pathway transitions from a matrix-dominated process to a filler-influenced mechanism, reflected by the earlier onset of degradation and the broadening and splitting of DTGA peaks.

This interplay between barrier-induced stabilization and filler-mediated catalytic effects accounts for the observed non-monotonic evolution of thermal stability with increasing ash content.

#### DTGA evolution and interfacial catalysis

The derivative thermogravimetric (DTGA) curves elucidate the transition from a matrix-governed to a filler-catalyzed degradation pathway. Pristine SR exhibits a relatively uniform, semi-systematic degradation characterized by a sharp primary peak. In contrast, the incorporation of silica ash progressively broadens the DTGA profiles and introduces multiple shoulders, signaling the activation of diverse, competing kinetic pathways.

**Fig. 12:**
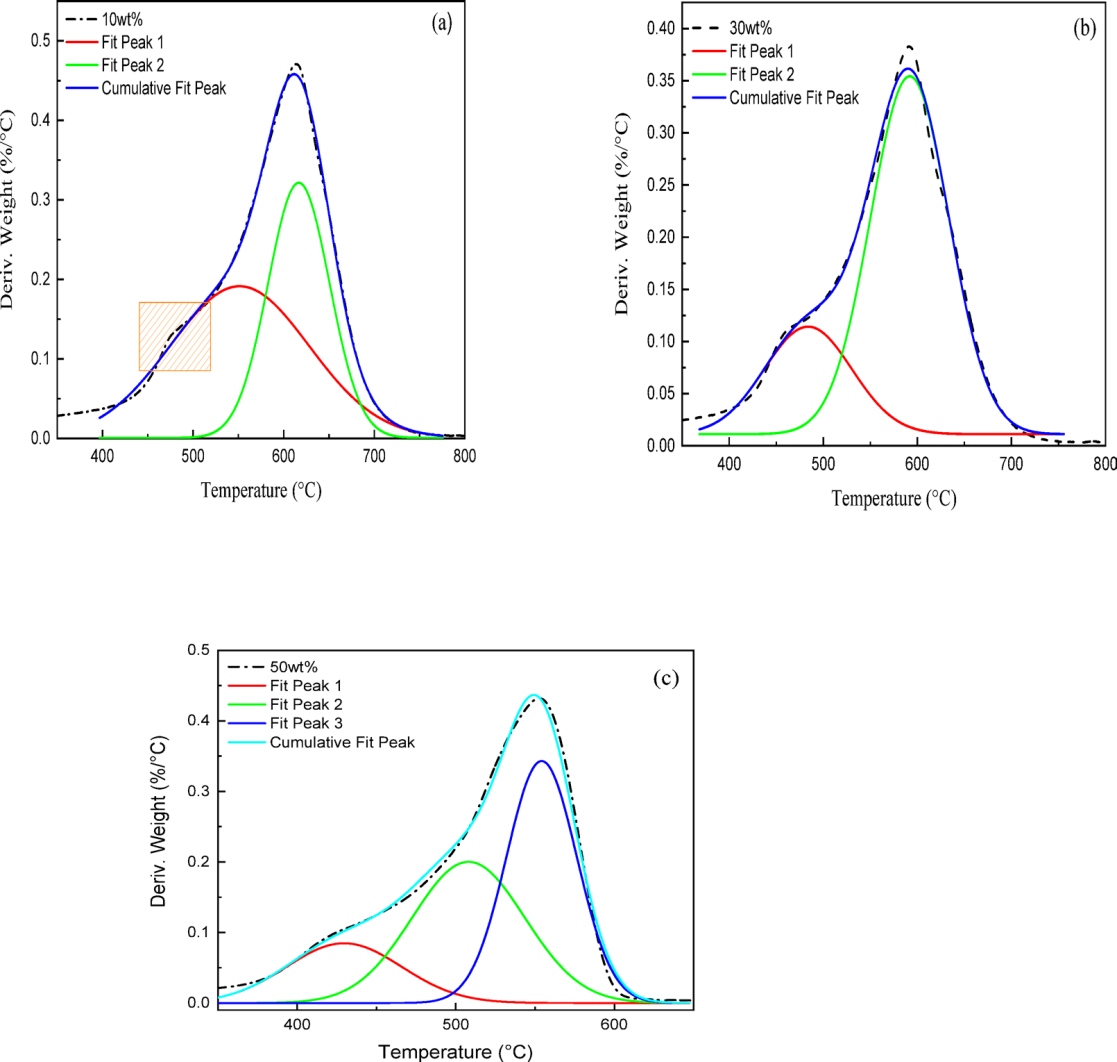
The deconvolution of DTGA peaks for the filled composites for silicone rubber loaded with (**a**) 19 wt%, (**b**) 30 wt%, and (**c**) 50 wt% silica ash.

The 10 wt% loading represents a thermal optimum where the peak degradation temperature (T max) is maximized. This suggests that optimal dispersion maximizes interfacial contact, providing a uniform stabilizing effect. At 30–50 wt%, however, particle agglomeration and the high density of active interfacial sites promote heterogeneous catalysis, shifting T max to lower temperatures and disrupting the coordinated degradation of the unfilled matrix. The derivative thermogravimetric (DTGA) profiles presented in Fig. 12b corroborate this interpretation. At low filler loadings, a relatively sharp and well-resolved degradation peak is observed, indicative of a homogeneous decomposition process. Conversely, higher ash contents result in peak broadening and the emergence of multiple shoulders, which reflect the activation of additional degradation pathways. This evolution in the peak structure is consistent with the growing influence of filler-related interactions and the onset of heterogeneous degradation kinetics at elevated filler loadings.

#### Deconvolution of complex degradation stages

To isolate specific degradation mechanisms, the overlapping DTGA peaks for 10, 30, and 50 wt% composites were deconvoluted, revealing a progressive increase in microstructural complexity:

- 10 wt% Ash: The profile is accurately modeled by two nearly equivalent stages (Fit Peak 1 & 2), suggesting a synchronized two-phase degradation with minimal catalytic interference.
- 30 wt% Ash: A shift occurs toward a dominant early-stage mechanism (Fit Peak 1), occurring near 480 °C. This highlights the activation of interfacial reactions such as side-group elimination or enhanced siloxane cleavage driven by moderate filler levels.
- 50 wt% Ash: The profile transforms into a trimodal structure. The emergence of three distinct fit peaks underscores the dominance of filler-catalyzed pathways, where abundant surface hydroxyls facilitate multiple simultaneous scission and recombination reactions, drastically lowering the overall thermal resistance of the composite.

Scientific Insight: This thermal “fingerprint” corroborates the mechanical trends observed in Sect.  4.3. The 10–15 wt% threshold serves as a dual optimum: providing enough interfacial area for mechanical reinforcement and thermal stabilization (barrier effect) without reaching the saturation limit where catalytic degradation and particle agglomeration (embrittlement) become the governing factors.

### Radiological characterization: photon attenuation dynamics

#### Energy-dependent evolution of attenuation cross-sections

**Fig. 13:**
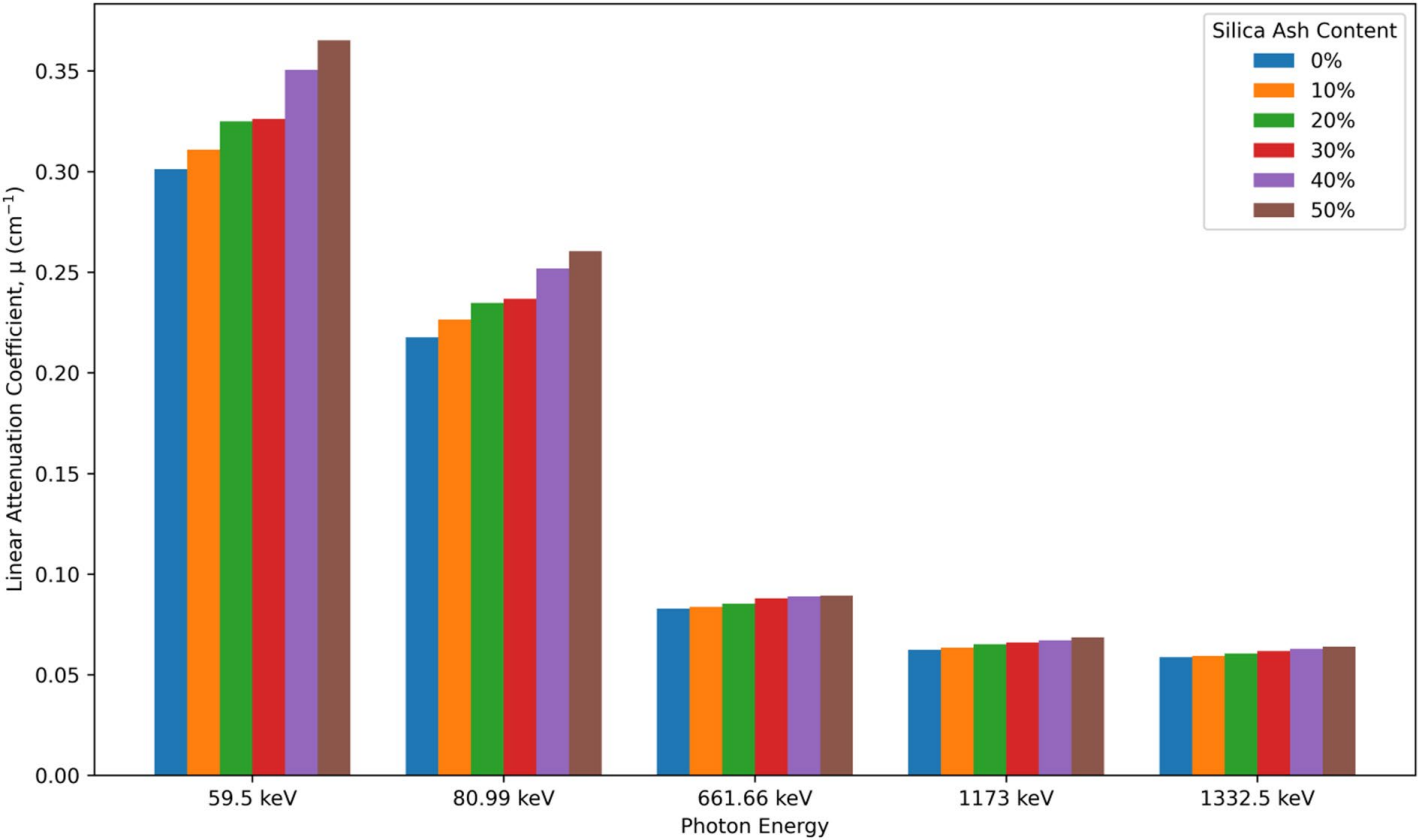
Variation of the linear attenuation coefficient (µ) as a function of photon energy for silicone rubber composites containing different silica ash concentrations (0–50 wt%).

The linear attenuation coefficients (µ) of the silica-ash-reinforced silicone rubber composites demonstrate a monotonically decreasing profile as a function of incident photon energy, revealing a distinct bimodal attenuation regime (Fig. 13). In the lower energy spectrum (59.5 to 661.66 keV), the composites exhibit a high magnitude attenuation response, with µ values for the pristine matrix descending sharply from 0.3011 cm⁻¹ to 0.0829 cm⁻¹, as shown in Table 5. This high-gradient decline is indicative of a transition from photoelectric-dominated absorption to incoherent (Compton) scattering. At 59.5 keV, the linear attenuation coefficient (µ) increases from 0.3011 ± 0.0034 cm⁻¹ (0 wt%) to 0.3651 ± 0.0025 cm⁻¹ (50 wt%) (*n* = 3), corresponding to an enhancement of approximately 21%. The relatively low standard deviation and coefficient of variation confirm that this improvement exceeds measurement uncertainty and is therefore statistically and physically meaningful. This enhancement is attributed to the increased contribution of the photoelectric effect at lower photon energies, which is highly sensitive to compositional changes and effective atomic number.

**Fig. 14:**
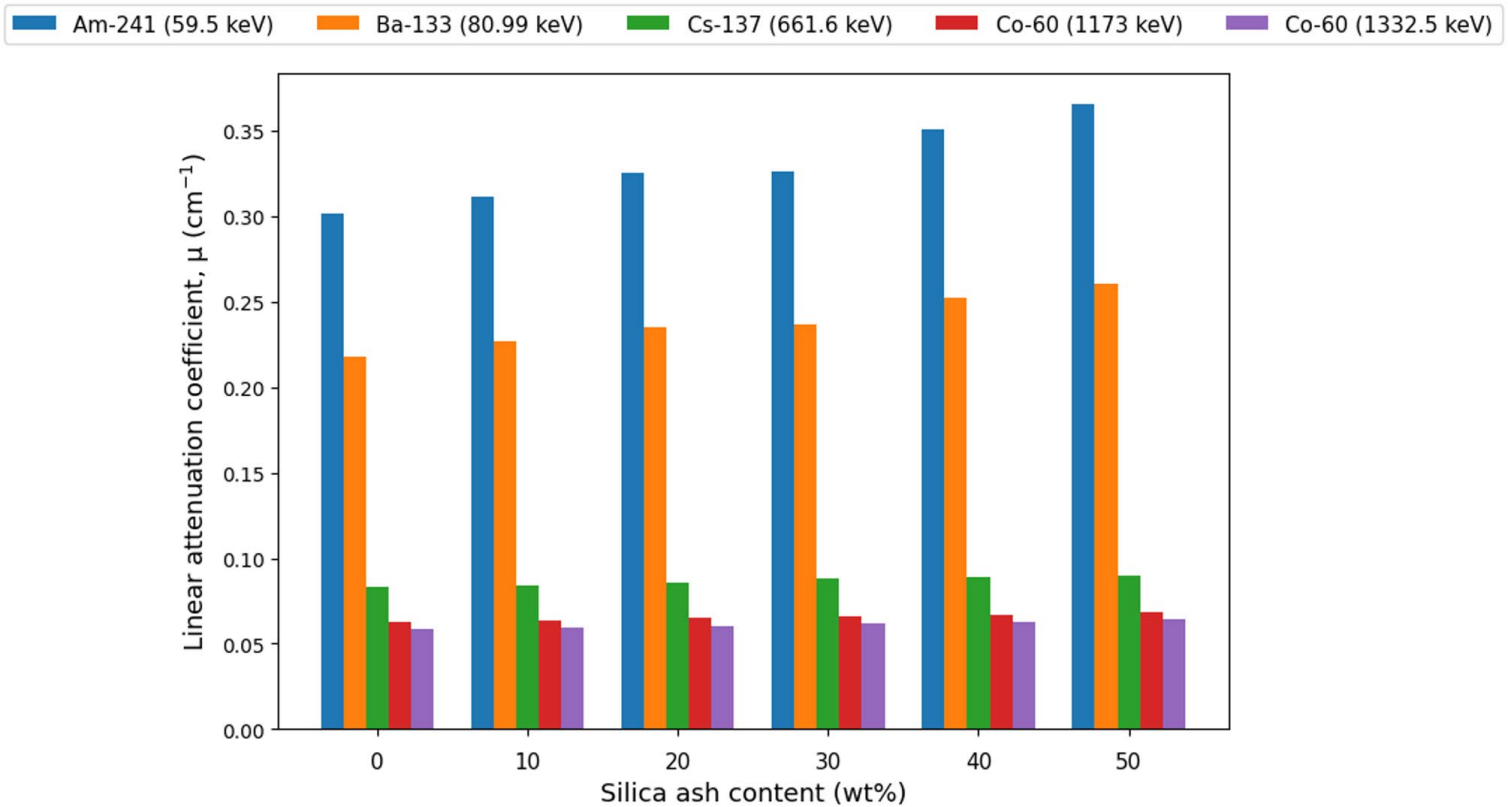
Linear attenuation coefficient (µ) versus silica ash content for different photon energies, illustrating the effect of filler loading on gamma-ray attenuation performance.

Beyond 661.66 keV, the attenuation curves plateau, with µ values converging toward a narrow range (0.0829 to 0.058 cm⁻¹), as the interaction probability becomes less sensitive to the chemical stoichiometry of the filler and more reliant on the bulk density of the composite, The variation of µ with silica ash content is shown in Fig. 14.

##### Low-energy regime: interaction mechanisms and zeff enhancement

At energies of 59.5 keV and 80.99 keV, the attenuation is fundamentally governed by the photoelectric effect, where the interaction probability (τ) is defined by the following relation:

13 $$\sigma _{{{\mathrm{pe}}}} \propto \frac{{{\mathrm{Z}}^{n} }}{{{\mathrm{E}}^{{3 - 4}} }}n\, \approx \,4 - 5$$

This extreme sensitivity to the effective atomic number (Z_eff_) explains the pronounced performance boost observed upon filler incorporation. The industrial silica ash introduces not only a silicon-rich framework (Z = 14) but also trace high-Z constituents (e.g., Fe, K, Ag) which disproportionately elevate the photoelectric cross-section compared to the pristine siloxane matrix.

Significantly, the 30 wt% silica ash composite achieves a mass attenuation coefficient (MAC) of 0.2812 cm².g^− 1^ at 59.5 keV, outperforming the 30 wt% Al₂O₃ composite (0.2239 cm².g^− 1^) by over 25%^2^. This enhancement is attributed to the superior Zeff of the waste-derived ash over pure alumina, proving that “waste” chemistry can provide more efficient interaction mechanisms with low-energy photons than technical-grade oxides.

##### High-energy regime: electron density and compton scattering behavior

As incident energy exceeds 300 keV, the contribution of the photoelectric effect becomes negligible, and Compton scattering emerges as the primary attenuation mechanism. In this regime, the interaction cross-section depends weakly on Z and is primarily a function of the electron density (ρ_e_) of the material.

At the ¹³⁷Cs energy level (661.65 keV), the 20 wt% silica ash composite maintains a competitive MAC of 0.0748 cm².g^− 1^. Remarkably, when extended to high-energy ⁶⁰Co sources (1173 and 1332.5 keV), the silica ash composite (0.0571 cm².g^− 1^) demonstrates high-performance stability, approaching the attenuation levels of high-density bismuth glasses like BS4 (Bismuth system oxides), (0.067 cm².g^− 1^)^5^.

This convergence indicates that while heavy-metal oxides like Bi₂O₃ (Z = 83) dominate the low-energy spectrum, the silica-ash-filled elastomers offer a weight-optimized shielding alternative in the scattering-dominated region. The high-energy performance is driven by the consistent distribution of the filler, ensuring a spatially uniform electron density that maximizes the probability of inelastic photon-electron collisions.

##### Interfacial synergy and physical validity

The seamless transition across these energy regimes validates the physical reliability of the experimental data. The synergistic relationship between the rough surface morphology and the elastomeric matrix previously observed via SEM-ensures that the macro-scale shielding properties are a direct reflection of the micro -scale filler dispersion. These results establish the processed silica ash as a high-performance, dual-regime enhancer, bridging the gap between low-cost industrial byproducts and high efficiency radiation-shielding materials.

##### Statistical comparison with XCOM data

A one-sample t-test was performed for each energy to compare experimental MAC values with theoretical values from the NIST XCOM. All p-values were greater than 0.05, indicating no statistically significant difference and confirming good agreement between experimental and theoretical results, The calculated p-values are provided in Supplementary Table S1.

**Table 5:** Experimental linear attenuation coefficient (µ), mass attenuation coefficient (µ/ρ) (mean ± standard deviation, *n* = 3), and percentage deviation from NIST XCOM values for silicone rubber/silica ash composites with different filler concentrations at various photon energies.

Ash Content (%)	Energy (keV)	µ (cm⁻¹) ± SD	CV%	MAC ± SD	CV%	XCOM MAC ( (cm².g^− 1^)	Δ Error %
0%	59.5	0.3011 ± 0.0034	1.1423	0.2737 ± 0.0031	1.1423	0.2752	0.5356
	80.99	0.2175 ± 0.0013	0.6082	0.1977 ± 0.0012	0.6082	0.2007	1.4903
	661.66	0.0829 ± 0.0014	1.6758	0.0754 ± 0.0013	1.6758	0.07727	2.4551
	1173	0.0623 ± 0.0013	2.0867	0.0566 ± 0.0012	2.0867	0.05876	3.6543
	1332.5	0.0587 ± 0.0011	1.9198	0.0534 ± 0.0010	1.9198	0.05506	3.1521
10%	59.5	0.3109 ± 0.0023	0.7495	0.2776 ± 0.0021	0.7495	0.2807	1.1128
	80.99	0.2263 ± 0.0019	0.8465	0.2021 ± 0.0017	0.8465	0.2047	1.2653
	661.66	0.0837 ± 0.0013	1.5623	0.0747 ± 0.0012	1.5623	0.07725	3.3619
	1173	0.0634 ± 0.0014	2.1912	0.0566 ± 0.0012	2.1912	0.05874	3.7028
	1332.5	0.0594 ± 0.0010	1.6060	0.0530 ± 0.0009	1.6060	0.05504	3.7669
20%	59.5	0.3248 ± 0.0019	0.5898	0.2849 ± 0.0017	0.5898	0.2875	0.881
	80.99	0.2347 ± 0.0016	0.6642	0.2059 ± 0.0014	0.6642	0.2085	1.2677
	661.66	0.0853 ± 0.0015	1.7270	0.0748 ± 0.0013	1.7270	0.0772	3.19319
	1173	0.0651 ± 0.0012	1.8687	0.0571 ± 0.0011	1.8687	0.0587	2.7529
	1332.5	0.0606 ± 0.0012	2.0075	0.0532 ± 0.0011	2.0075	0.055	3.4548
30%	59.5	0.3262 ± 0.0024	0.7490	0.2812 ± 0.0021	0.7490	0.2852	1.4156
	80.99	0.2367 ± 0.0016	0.6599	0.2041 ± 0.0013	0.6599	0.2064	1.144
	661.66	0.0879 ± 0.0014	1.5805	0.0758 ± 0.0012	1.5805	0.0772	1.8061
	1173	0.0660 ± 0.0016	2.3667	0.0569 ± 0.0013	2.3667	0.0587	3.1312
	1332.5	0.0618 ± 0.0008	1.2843	0.0533 ± 0.0007	1.2843	0.055	3.21418
40%	59.5	0.3505 ± 0.0030	0.8677	0.2970 ± 0.0026	0.8677	0.2997	0.8825
	80.99	0.2518 ± 0.0019	0.7608	0.2134 ± 0.0016	0.7608	0.2153	0.8902
	661.66	0.0889 ± 0.0014	1.5627	0.0753 ± 0.0012	1.5627	0.0771	2.4077
	1173	0.0671 ± 0.0015	2.1954	0.0569 ± 0.0012	2.1954	0.0586	3.1185
	1332.5	0.0628 ± 0.0011	1.6852	0.0532 ± 0.0009	1.6852	0.0549	3.19995
50%	59.5	0.3651 ± 0.0025	0.6891	0.3077 ± 0.0054	1.7649	0.3073	0.9863
	80.99	0.2604 ± 0.0019	0.7112	0.2170 ± 0.0015	0.7112	0.2196	1.1979
	661.66	0.0893 ± 0.0015	1.6722	0.0744 ± 0.0012	1.6722	0.07713	3.5609
	1173	0.0685 ± 0.0013	1.8978	0.0571 ± 0.0011	1.8978	0.05862	2.6356
	1332.5	0.0639 ± 0.0011	1.7636	0.0533 ± 0.0009	1.7636	0.05493	3.0575

**Table 6:** Half-value layer (HVL), tenth-value layer (TVL), mean free path (MFP), for silicone rubber/silica ash composites with different filler concentrations at various photon energies.

Ash content	Energy (KeV)	HVL (cm)	TVL (cm)	MFP (cm)	Density (gcm^− 3^)
0%	59.5	2.30199	7.64706	3.32107	1.1
	80.99	3.1864	10.585	4.59711	
	661.66	8.3551	27.755	12.0539	
	1173	11.1157	36.925	16.0366	
	1332.5	11.8052	39.216	17.0313	
10%	59.5	2.22931	7.40562	3.21622	1.12
	80.99	3.06161	10.1704	4.41697	
	661.66	8.2807	27.50804	11.9465	
	1173	10.9260	36.2956	15.7629	
	1332.5	11.6677	38.7595	16.833	
20%	59.5	2.13349	7.08733	3.07798	1.14
	80.99	2.95315	9.81016	4.2604	
	661.66	8.12534	26.9918	11.7223	
	1173	10.6414	35.3502	15.3524	
	1332.5	11.4348	37.9856	16.4969	
30%	59.5	2.12482	7.05851	3.06547	1.16
	80.99	2.92818	9.72720	4.22447	
	661.66	7.88098	26.1800	11.3698	
	1173	10.4983	34.8746	15.1458	
	1332.5	11.2135	37.2506	16.1777	
40%	59.5	1.9773	6.56845	2.8526	1.18
	80.99	2.7526	9.14405	3.97121	
	661.66	7.79622	25.8984	11.2475	
	1173	10.3279	34.3085	14.90003	
	1332.5	11.0300	36.640	15.9129	
50%	59.5	1.89820	6.30571	2.73853	1.2
	80.99	2.66185	8.84247	3.84023	
	661.66	7.75562	25.7636	11.1889	
	1173	10.1134	33.5960	14.5905	
	1332.5	10.83712	36.00016	15.6346	

#### Quantum-mechanical basis of energy-dependent attenuation

The transition from a photoelectric-dominated regime to a Compton-dominated regime is fundamentally a consequence of the wave-particle duality of the incident gamma radiation and its interaction with the atomic architecture of the composite.

##### Wavelength-scale interaction and photoelectric absorption

At low incident energies (59.5 to 80.99 keV), the photon wavelength (λ) is relatively large and comparable to the dimensions of atomic orbitals. This allows for strong electromagnetic coupling with the tightly bound inner-shell (K-shell) electrons, which possess the highest binding energies. Because the incident photon energy in this regime is near the binding energy of these electrons, the probability of complete absorption is maximized, resulting in the high initial mass attenuation coefficients (MAC) observed for both the pristine silicone rubber and the silica-ash-filled samples.

##### High-energy stochasticity and compton scattering

As photon energy increases toward the ¹³⁷Cs (661.66 keV) and ⁶⁰Co (1173-1332.5 keV) levels, the wavelength contracts significantly, becoming much smaller than the Bohr radius of the atom. In this high-momentum state:

- **Quasi-Free Electron Interaction**: The incident energy far exceeds the binding energy of outer-shell (valence) electrons. Consequently, these electrons are treated as “quasi-free,” and the interaction shifts from total absorption to inelastic scattering (Compton effect). The photon transfers only a fraction of its energy to the electron and continues along a deflected path.
- **Electronic Transparency and Penetration**: The reduction in wavelength diminishes the effective “target size” (cross-section) for absorption. This allows high-energy photons to penetrate deeper into the electronic cloud, bypassing outer-shell interactions with a higher probability of reaching the denser inner-shell environment or the nucleus.
- **Mean Free Path and Convergence**: This increased penetration depth is reflected in the systematic decrease of the linear attenuation coefficient (µ) and the convergence of MAC values at high energies. In this regime, the attenuation is governed strictly by the volumetric electron density of the silica-ash filler rather than specific atomic number (Z)-effects, explaining why the performance gap between the low-Z ash and high-Z bismuth glass narrows significantly.

#### Implications for shielding thickness: HVL, TVL, and MFP

**Fig. 15:**
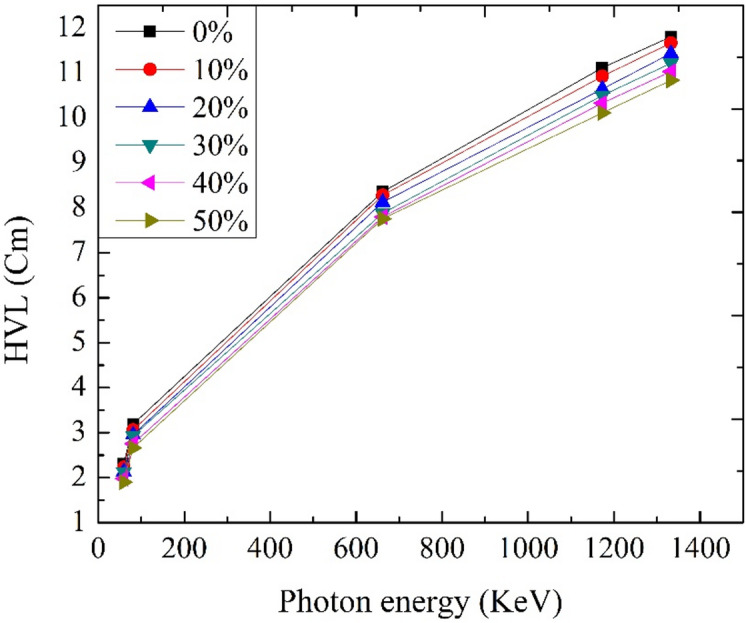
Dependence of half-value layer (HVL) on photon energy for silicone rubber/silica ash composites at various filler concentrations.

**Fig. 16:**
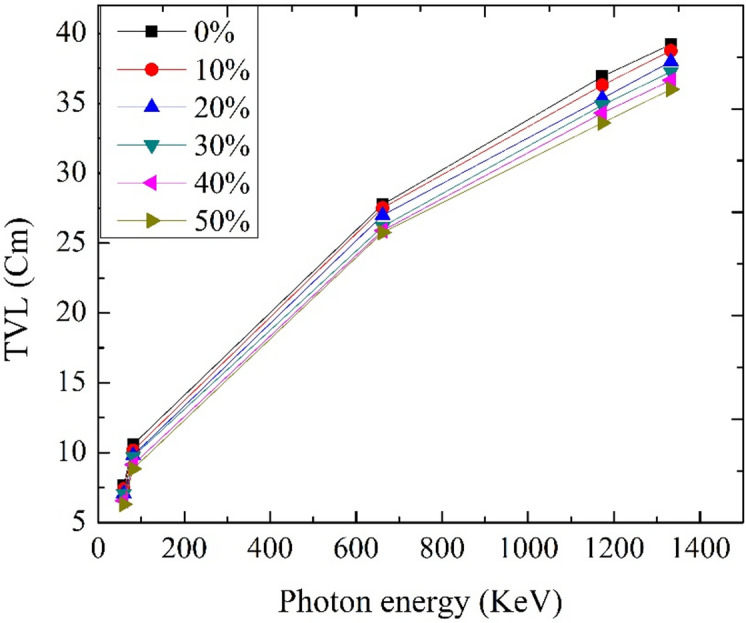
Dependence of tenth-value layer (TVL) on photon energy for silicone rubber/silica ash composites at various filler concentrations.

**Fig. 17:**
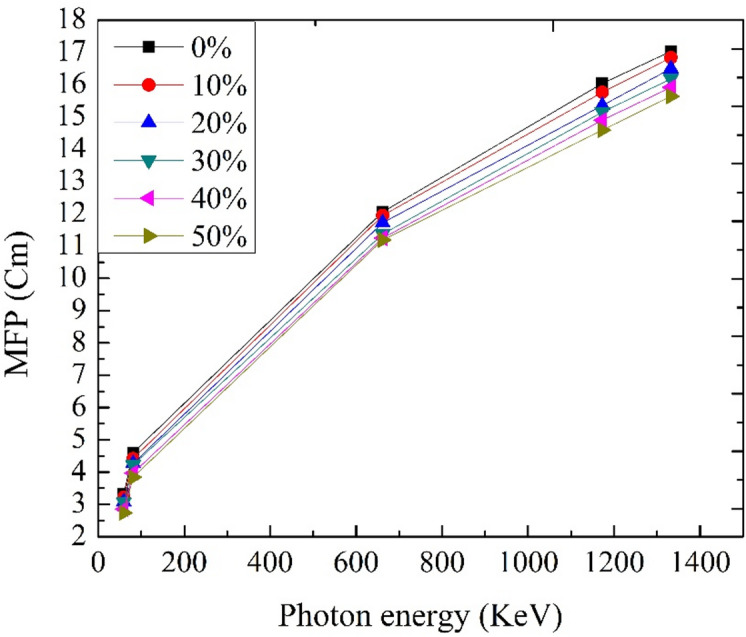
Variation of mean free path (MFP) with photon energy for silicone rubber/silica ash composites at different silica ash loadings.

The improvement in attenuation efficiency with increasing silica ash content is further reflected in the derived shielding parameters HVL, TVL, and MFP are summarized in Table 6. As shown in Figs. 15, 16 and 17, For a fixed photon energy, all three parameters decrease systematically with increasing filler concentration, indicating that thinner material layers are required to achieve equivalent attenuation.

At 59.5 keV, the reduction in HVL from approximately 2.30 cm (0 wt%) to 1.90 cm (50 wt%) highlights the strong influence of silica ash loading in the photoelectric-dominated region. Similar, albeit more gradual, reductions are observed at higher energies, confirming that silica ash enhances shielding performance across the entire investigated energy spectrum.

##### Experimental-theoretical consistency

The relatively small standard deviations indicate good experimental repeatability. The excellent agreement between experimental mass attenuation coefficients and NIST XCOM predictions, with deviations generally below **3.76%**, further supports the physical validity of the observed trends. This agreement confirms that the enhancement in attenuation with silica ash loading arises from well-understood photon-matter interaction mechanisms rather than experimental artifacts.

#### Comparative analysis

##### Silica ash vs. alumina (Al_2_O_3_)

The radiological performance of the silica-ash-reinforced silicone rubber was benchmarked against conventional Al_2_O_3_ filled systems. A direct comparison of mass attenuation coefficients MAC at 59.5 keV reveals a significant superiority for the silica ash filler. For instance, at a 30 wt% loading, the silica ash composite achieves a MAC 0.2812 cm².g^-1^, whereas the corresponding Al_2_O_3_ composite yields only 0.2239 cm².g^-1^^2^.

This discrepancy highlights a critical advantage: the silica ash provides approximately 25.6% higher attenuation efficiency than alumina at the same concentration. Remarkably, even at a low 10 wt% loading, the silica ash composite (0.2776 cm².g^-1^) outperforms the 40 wt% composite Al_2_O_3_ (0.2233 cm².g^-1^).

##### Physical mechanism for enhanced performance

This enhanced performance is dictated by the elemental composition of the fillers:

- Atomic Number (Z) Advantage: While Aluminum has an atomic number of Z = 13, the primary constituent of the ash, Silicon, has Z = 14. Given that the photoelectric cross-section (τ) is highly sensitive to Z (τ proportional to Z^4–5^), the slightly higher Z of Silicon, coupled with the trace high Z industrial constituents (Fe, K, Ag) identified in the EDX/XRF analysis, significantly amplifies the interaction probability.
- Filler-Matrix Synergy: The data suggests that silica ash maintains a more effective electron density for shielding than pure alumina. In the alumina-filled system, µ values actually show a slight decrease as filler concentration increases at 59.5 keV (from 0.2316 for pure SR to 0.2233 for 40 wt% filler). In contrast, the silica ash composites consistently increase the shielding capability of the matrix.

##### Economic and environmental impact

Beyond radiological superiority, the use of silica ash represents a **“Waste to Wealth”** paradigm. By replacing processed, high-cost Al_2_O_3_ with industrial waste-derived ash, the manufacturing cost of radiation-shielding elastomers is drastically reduced while simultaneously addressing industrial waste management challenges.

##### Silica ash vs. bismuth-oxide systems

A comparative analysis of the mass attenuation coefficients MAC reveals distinct performance regimes based on photon energy. At the low-energy threshold 59.5 keV, the Bismuth glass system BS4 (20% Bi_2_O_3_) exhibits a markedly higher MAC of 1.3948 cm².g^-1^ compared to 0.28 cm².g^-1^ for the 20 wt% silica ash composite^5^. This behavior is expected, as the photoelectric effect scales with the atomic number, allowing the heavy Bismuth atoms (Z = 83) to disproportionately dominate the interaction probability compared to the Silicon-based (Z = 14) ash filler.

However, as the system transitions into the Compton scattering-dominated region, a significant performance convergence is observed:

- Intermediate Energy (661 keV): At the ^137^Cs energy level, the gap between high-Z glass and the elastomeric composite narrows significantly. The MAC for the Bismuth-rich BS4 glass is 0.101 cm².g^-1^, while the 20 wt% silica ash composite maintains a highly competitive value of 0.0748 cm^2^.g^-1^, The attenuation gap narrows from nearly 460% at low energy to just **25.9%** in the intermediate energy range.
- High Energy (^60^Co 1173 and 1332 keV): In the high-energy regime, the influence of the atomic number further diminishes in favor of electron density. For the BS4 system, MAC values reach 0.067 cm².g^-1^ Remarkably, the 20 wt% silica ash composite achieves a MAC of 0.0571 cm^2^.g^-1^, The attenuation gap just **10.7%** in the High energy range.

##### The case for lightweight sustainability

While Bismuth glasses provide higher absolute attenuation, the silica ash composites offer a superior weight-to-shielding ratio. The density of the BS4 glass system 4.631 g.cm^-3^ is nearly four times higher than that of the 20 wt% silica ash composite 1.14 g.cm^-3^.

From a practical engineering standpoint, the silica ash filler provides sufficient shielding for high-energy applications while avoiding the mechanical embrittlement and high costs associated with heavy-metal oxides. By utilizing a “waste-to-wealth” byproduct, this study demonstrates a sustainable path toward developing flexible, lightweight radiation shields that offer significant protection without the mass penalty of traditional high-Z materials.

The results demonstrate that silica ash, despite being a low-toxicity, light and industrial waste-derived filler, can effectively enhance gamma-ray shielding performance when incorporated at sufficient concentrations. This highlights the potential of silica ash-filled silicone rubber composites as sustainable, flexible, and multifunctional shielding materials for radiation protection applications.

#### Lead-equivalent thickness analysis and benchmarking against conventional shielding

**Table 7:** Lead-equivalent thickness ratio and HVL benchmarking for 50 wt% Silica-Ash Filled Silicone Rubber.

Radiation source	Photon energy (MeV)	HVL_Pb_ (mm)	HVL_SR_ (mm)	Pb-Equivalent Ratio (x_SR_/x_Pb_)
^241^Am	0.0595	0.1	18.98	184
^133^Ba	0.081	0.31	26.65	87
^137^Cs	0.6616	5.55	77.87	14
^60^Co	1.173	9.63	101.17	10.5
^60^Co	1.3325	11	108.28	9.8

To evaluate the practical shielding efficacy of the 50 wt% silica ash composite, a comparative analysis was conducted against a pure lead (Pb) benchmark (ρ = 11.34 g.cm^− 3^). The lead-equivalent thickness (x_Pb_) was determined by equating photon transmission via the Beer-Lambert relation.

14 $$\mu _{{{\mathrm{SR}}}} {\mathrm{x}}_{{{\mathrm{SR}}}} \, = \,\mu _{{{\mathrm{Pb}}}} {\mathrm{x}}_{{{\mathrm{Pb}}}}$$

, where µ represents the linear attenuation coefficients derived from experimental and NIST-XCOM data. For engineering design purposes, the half-value layer (HVL) the thickness required to attenuate incident intensity by 50% was calculated as the primary performance metric, The lead-equivalent thickness and HVL benchmarking results are presented in Table 7.

##### Energy-dependent efficiency and interaction transitions

The data reveals a profound convergence in shielding efficiency as the incident energy increases. In the photoelectric-dominated regime (≤ 0.1 MeV), the high-Z discrepancy between lead (Z = 82) and the silica ash (Z_eff_ ≈ 14) results in thickness ratios exceeding two orders of magnitude. However, as Compton scattering becomes the governing mechanism (> 0.6 MeV), the interaction cross-section becomes a function of electron density rather than atomic number.

In this high-energy regime, the performance gap narrows significantly. At 1.33 MeV, the Pb-equivalent ratio drops to 9.8, implying that a roughly 1 cm layer of the 50 wt% ash composite provides protection equivalent to 1 mm of lead. This transition validates the use of silica-ash-reinforced elastomers for high-energy applications where the electron density provided by the filler compensates for its lower atomic number.

##### Engineering and sustainability implications

While lead maintains a lower volumetric footprint, the silica ash composite offers a superior weight-to-flexibility ratio and addresses the critical environmental challenges of lead toxicity. By utilizing a “waste-to-wealth” industrial byproduct, this material provides a sustainable, non-toxic, and formable alternative for wearable shielding or complex geometries where rigid lead sheets are functionally and ecologically unviable.

### Mechanical performance vs. gamma-ray shielding

**Fig. 18:**
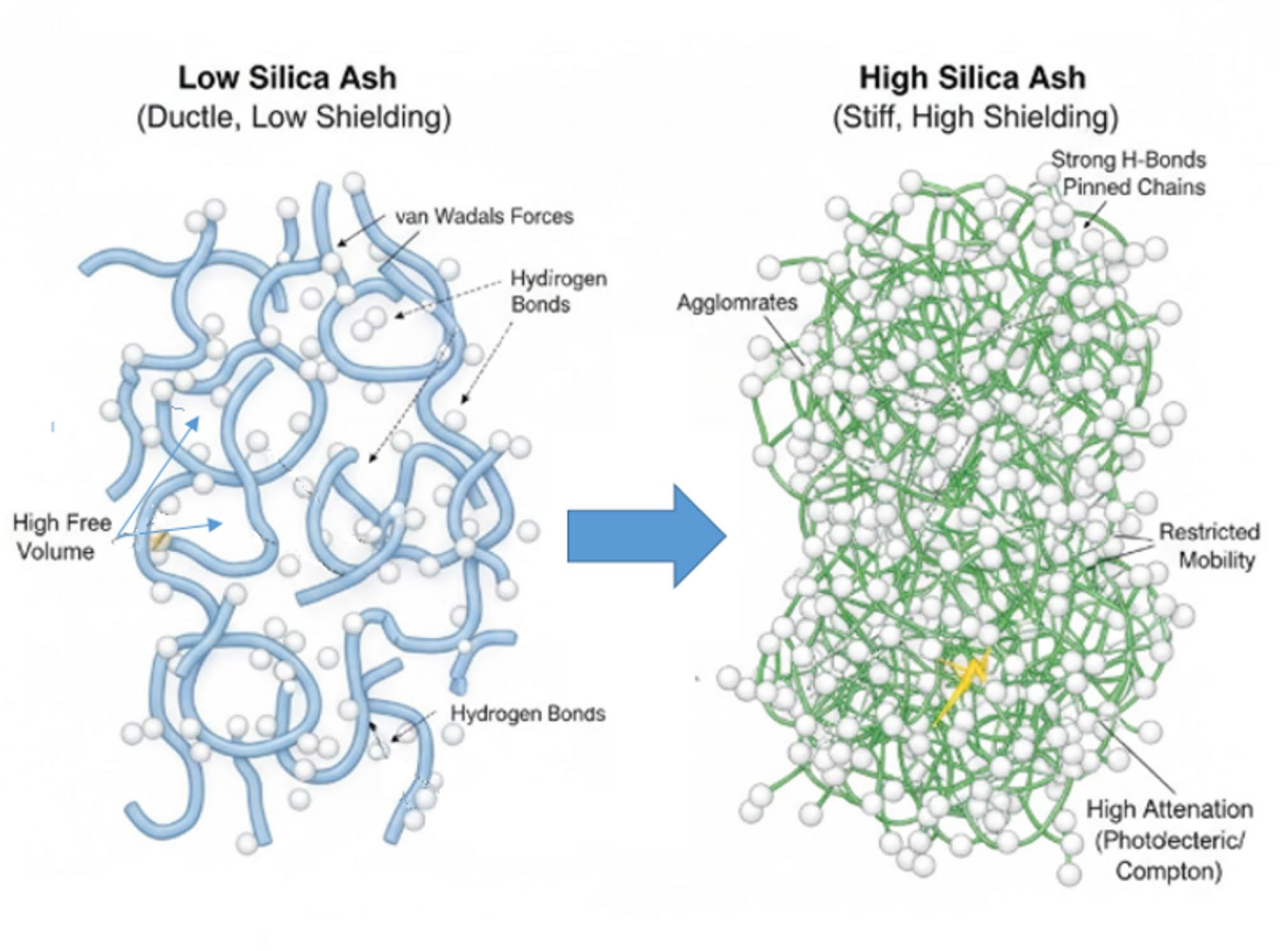
Schematic representation of the microstructural transition in silicone rubber with increasing silica ash content. Low filler loading yields a flexible, high free-volume network, whereas high loading induces chain immobilization, dense filler networks, and enhanced gamma-ray attenuation via photoelectric and Compton interactions.

A key strength of the present work lies in the simultaneous optimization of radiation shielding and mechanical integrity, which is rarely achieved in flexible polymer-based shielding materials. From the radiation results, increasing silica ash content leads to a systematic increase in linear attenuation coefficient and a corresponding reduction in HVL, TVL, and MFP, driven by increased density, electron density, and effective atomic number contributions, Conversely, the mechanical results reveal that increasing filler content enhances stiffness but reduces ductility and toughness beyond an optimal threshold. This leads to an important design trade-off:

- High filler contents (≥ 30 wt%) maximize gamma-ray attenuation efficiency but at the expense of flexibility and toughness.
- Intermediate filler contents (≈ 15–20 wt%) provide a balanced performance, combining: significant enhancement in gamma-ray attenuation, improved tensile strength and stiffness, high energy absorption capability. Such a balance is particularly desirable for flexible radiation shielding applications, including wearable protective materials, medical radiation barriers, and shielding components requiring mechanical compliance, The microstructural transition is illustrated in Fig. 18.

## Conclusion

This study pioneers sustainable polydimethylsiloxane (PDMS) elastomers reinforced with 0–50 wt% silica ash an upcycled siliceous byproduct as intrinsically flexible gamma-ray shields, synergistically optimizing photonic attenuation, viscoelastic reinforcement, and thermal resilience. Narrow-beam dosimetry across diagnostic-to-isotopic spectra (59.5-1332.5 keV) unveils monotonic escalation in linear attenuation coefficients (µ), culminating at 0.3651 cm⁻¹ (50 wt% ash, 59.5 keV), with half-value layers contracting from 2.30 cm to 1.90 cm. This derives from a mechanistic pivot: Z_eff_ augmented photoelectric dominance (< 100 keV) yields to ρ-modulated Compton scattering (≥ 661 keV), corroborated by mass attenuation coefficients aligning within 3.7% of NIST XCOM cross-sections, affirming compositional fidelity and beam-collimation rigor.

Hierarchical nanofiller integration evidenced by SEM/EDX/XRF induces interfacial siloxane bridging, restricting Gaussian chain conformations and elevating Young’s modulus from 0.026 to 0.157 MPa (40 wt%). Mechano-elastic optima emerge at 15 wt% (tensile strength: 0.357 MPa; toughness: 0.591 MJ·m⁻³), beyond which percolation-driven agglomeration precipitates embrittlement via dewetting-induced stress risers. Thermogravimetric dissection reveals a 10 wt% stabilization apex, supplanted by Lewis-acidic SiO₂ catalysis of depolymerizing Si-O scission at higher loadings, despite enhanced char yields.

Critically, 10–20 wt% silica ash loadings calibrate a triadic synergy: superior mass-specific attenuation (10 to 14× lead efficiency in Compton domain), retained hyper-elasticity (> 100% strain), and closed-loop sustainability repurposing siliceous waste to supplant Pb/Bi paradigms. These attributes position ash-PDMS composites as transformative for conformable medical aprons, nuclear gloves, wearable dosimetry, and extraterrestrial habitats, redefining elastomeric radiation resilience.

## Supplementary Information

Below is the link to the electronic supplementary material.

Supplementary Material 1

Supplementary Material 2

## Data Availability

All data generated or analysed during this study are included in this published article and its supplementary information files.
