# Correction: Optimization of linear attenuation coefficients and characterization of mechanical and thermal properties in silica ash-reinforced PDMS composites

**DOI:** 10.1038/s41598-026-61291-2

**Published:** 2026-07-07

**Authors:** Maged Mostafa, S. S. Ibrahim, Sherif A. Khairy, Ahmed M. El-khatib

**Affiliations:** 1 Physics Department, Faculty of Science, Cairo University, Giza, Egypt; 2 Physics Department, Faculty of Science, Alexandria University, Alexandria, 21511 Egypt

Correction to: *Scientific Reports* 10.1038/s41598-026-58992-z, published online 29 June 2026

The original version of this Article contained errors in Equations 11 and 12, where

Equation 11 was incorrectly given as:

$${U}_{t}={\int}_{0}^{?}\sigma \left(?\right)d?$$

Equation 11 now reads:

$${U}_{t}={\int}_{0}^{\epsilon}\sigma \left(\epsilon\right)d\epsilon$$

Equation 12 was incorrectly given as:

$${U}_{t}=\sum_{i}^{n-1}\frac{\left({\sigma}_{i}+{\sigma}_{i+1}\right)}{2}.\left({?}_{i+1}-{?}_{i}\right)$$

Equation 12 now reads:

$${U}_{t}=\sum_{i}^{n-1}\frac{\left({\sigma}_{i}+{\sigma}_{i+1}\right)}{2}.({{\epsilon}}_{i+1}-{{\epsilon}}_{i})$$

The original Article has been corrected.

