# Supplementary material for: Optimization of linear attenuation coefficients and characterization of mechanical and thermal properties in silica ash-reinforced PDMS composites
Source: Sci Rep. 2026 Jun 29;16:19790. doi: 10.1038/s41598-026-58992-z (PMC13315010; doi:10.1038/s41598-026-58992-z)
Supplement: Supplementary file 2 — Supplementary Material 2 [file 41598_2026_58992_MOESM2_ESM.docx]

| **Ash (%)** | **Energy (keV)** | **Mean MAC (cm²/g)** | **SD** | **XCOM (cm²/g)** | **p-value** |
| --- | --- | --- | --- | --- | --- |
| 0 | 59.5 | 0.2737 | 0.0031 | 0.2752 | 0.386 |
| 0 | 80.99 | 0.1977 | 0.0012 | 0.2007 | 0.051 |
| 0 | 661.66 | 0.0753 | 0.0013 | 0.07727 | 0.119 |
| 0 | 1173 | 0.0566 | 0.0012 | 0.05876 | 0.09 |
| 0 | 1332.5 | 0.0533 | 0.001 | 0.05506 | 0.098 |
| 10 | 59.5 | 0.2776 | 0.002 | 0.2807 | 0.122 |
| 10 | 80.99 | 0.2021 | 0.0016 | 0.2047 | 0.114 |
| 10 | 661.66 | 0.0747 | 0.0012 | 0.07725 | 0.067 |
| 10 | 1173 | 0.0566 | 0.0011 | 0.05874 | 0.093 |
| 10 | 1332.5 | 0.053 | 0.0008 | 0.05504 | 0.054 |
| 20 | 59.5 | 0.2849 | 0.0017 | 0.2875 | 0.112 |
| 20 | 80.99 | 0.2059 | 0.0014 | 0.2085 | 0.083 |
| 20 | 661.66 | 0.0748 | 0.0013 | 0.0772 | 0.085 |
| 20 | 1173 | 0.0571 | 0.001 | 0.0587 | 0.117 |
| 20 | 1332.5 | 0.0532 | 0.001 | 0.055 | 0.097 |
| 30 | 59.5 | 0.2812 | 0.0019 | 0.2852 | 0.081 |
| 30 | 80.99 | 0.2041 | 0.0013 | 0.2064 | 0.094 |
| 30 | 661.66 | 0.0758 | 0.0012 | 0.0772 | 0.184 |
| 30 | 1173 | 0.0569 | 0.0013 | 0.0587 | 0.14 |
| 30 | 1332.5 | 0.0533 | 0.0007 | 0.055 | 0.052 |
| 40 | 59.5 | 0.297 | 0.0025 | 0.2997 | 0.215 |
| 40 | 80.99 | 0.2134 | 0.0017 | 0.2153 | 0.185 |
| 40 | 661.66 | 0.0753 | 0.0012 | 0.0771 | 0.123 |
| 40 | 1173 | 0.0569 | 0.0012 | 0.0586 | 0.137 |
| 40 | 1332.5 | 0.0532 | 0.0009 | 0.0549 | 0.08 |
| 50 | 59.5 | 0.3077 | 0.0055 | 0.3073 | 0.903 |
| 50 | 80.99 | 0.217 | 0.0016 | 0.2196 | 0.105 |
| 50 | 661.66 | 0.0744 | 0.0012 | 0.07713 | 0.061 |
| 50 | 1173 | 0.0571 | 0.0011 | 0.05862 | 0.128 |
| 50 | 1332.5 | 0.0533 | 0.0008 | 0.05493 | 0.085 |

Supplementary Table S1

Statistical comparison (one-sample t-test) between experimental MAC and theoretical values from NIST XCOM

All p-values were obtained using a one-sample Student’s t-test comparing experimental mean MAC values with theoretical data from the NIST XCOM. The significance level was set at p < 0.05. No statistically significant differences were observed.

**Table S2**. One-way ANOVA results for mechanical properties as a function of silica ash content

| **Property** | **Source** | **DF** | **Sum of Squares (SS)** | **Mean Square (MS)** | **F-value** | **P-value** |
| --- | --- | --- | --- | --- | --- | --- |
| **Tensile Strength (MPa)** | Ash Content | 6 | 0.056488 | 0.009415 | 72.58 | < 0.001 |
|  | Error | 14 | 0.001816 | 0.00013 | - | - |
|  | Total | 20 | 0.058304 | - | - | - |
| **Young’s Modulus (MPa)** | Ash Content | 6 | 0.046662 | 0.007777 | 107.35 | < 0.001 |
|  | Error | 14 | 0.001014 | 0.000072 | - | - |
|  | Total | 20 | 0.047676 | - | - | - |
| **Strain at Rupture** | Ash Content | 6 | 36.1169 | 6.01949 | 272.2 | < 0.001 |
|  | Error | 14 | 0.3096 | 0.02211 | - | - |
|  | Total | 20 | 36.4265 | - | - | - |
| **Toughness (MJ/m³)** | Ash Content | 6 | 0.611035 | 0.101839 | 312.98 | < 0.001 |
|  | Error | 14 | 0.004555 | 0.000325 | - | - |
|  | Total | 20 | 0.61559 | - | - | - |

**Table S2.** One-way ANOVA results showing the effect of silica ash content on mechanical properties. All properties exhibit statistically significant differences (p < 0.001).
